# Heptapharmacological activity of Quercetin-3-O-phosphate against lung cancer pathways

**DOI:** 10.1371/journal.pone.0349042

**Published:** 2026-07-10

**Authors:** Maryam Abdulrahman Alahdal, Hadil Alahdal, Najat Binothman, Majidah Aljadani, Nawal Helmi, Maryam A. AL-Ghamdi, Alaa Hamed Habib, Misbahuddin Rafeeq, Tahani Bakhsh, Hadeel A. Alsufyani, Abeer Al Tuwaijri

**Affiliations:** 1 College of Science, Department of Biology, Umm Al-Qura University, Makkah, Saudi Arabia; 2 Department of Biology, College of Science, Princess Nourah bint Abdulrahman University, Riyadh, Saudi Arabia; 3 Department of Chemistry, College of Sciences and Arts, King Abdulaziz University, Rabigh, Saudi Arabia; 4 Vaccine and Immunotherapy Unit, King Fahad Medical Research Center, King Abdulaziz University, Jeddah, Saudi Arabia; 5 Department of Biochemistry, College of Science, University of Jeddah, Jeddah, Saudi Arabia; 6 Biochemistry Department, Faculty of Science, King Abdulaziz University, Jeddah, Saudi Arabia; 7 Experimental Biochemistry Unit, King Fahd Medical Research Center, King Abdulaziz University, Jeddah, Saudi Arabia; 8 Department of Physiology, Faculty of Medicine, King Abdulaziz University, Jeddah, Saudi Arabia; 9 Department of Pharmacology, Basic Medical Sciences Division, College of Medicine, Dhofar University, Oman; 10 Department of Biological Sciences, College of Science, University of Jeddah, Jeddah, Saudi Arabia; 11 Department of Clinical Physiology, Faculty of Medicine, King Abdulaziz University, Jeddah, Saudi Arabia; 12 Medical Genomics Research Department, King Abdullah International Medical Research Center (KAIMRC), Ministry of National Guard Health Affairs (MNGH), Saudi Arabia; 13 Clinical Laboratory Sciences Department, College of Applied Medical Sciences, King Saud Bin Abdulaziz University for Health Sciences, Riyadh, Saudi Arabia; University of Sahiwal, PAKISTAN

## Abstract

Lung cancer is one of the leading causes of cancer-related deaths globally, with smoking being the primary risk factor, though environmental exposures and genetic mutations also play significant roles. Lung cancer arises when abnormal lung cells grow uncontrollably, leading to over 2 million new cases and nearly 1.8 million deaths globally. Drug resistance, especially to targeted therapies, is a key challenge, as tumours often adapt and become unresponsive. Multitargeted drug design, which targets multiple pathways involved in tumour growth, offers a promising solution to overcome resistance and improve treatment efficacy. In this study, we identified several lung cancer–associated proteins: CK2, Ran–Importin β complex, HNGF, Human Survivin, CRK-II adaptor protein, AKR1B10, and tRNA dihydrouridine synthase 2, with respective PDB IDs 1JWH, 1IBR, 1SG1, 1XOX, 2DVJ, 4XZL, and 4XP7. We conducted molecular docking using DrugBank’s library, employing HTVS, SP, and XP, followed by pose processing with MM/GBSA. Our multitarget docking analysis identified *Quercetin-3-O-Phosphate* as the top compound, a quercetin derivative found in fruits and vegetables such as onions, apples, berries, broccoli, and citrus. The compound showed docking and MM/GBSA scores ranging from −5.835 to −11.627 Kcal/mol and −14.28 to −47.07 Kcal/mol, respectively, and the key interacting residues with their counts include 10ASP, 9LEU, 9LYS, 7ALA, 7ARG, 7TYR, 6GLN, 5ASN, 5GLU, 5ILE, and 5VAL. We also performed and analysed DFT and pharmacokinetics in detail, which supported its further evaluation as it met all the required criteria. We conducted a 5 ns WaterMap simulation, which confirmed the interactions and highlighted key hydration sites that support the complex’s stability. A 100 ns MD simulation using the TIP3P water model showed minimal deviation and fluctuations, indicating stable interactions and the trajectories used for MM/GBSA calculations, assessing binding free, which reinforced the stability of the complexes and helped rank the best candidates. All results indicate that Quercetin-3-O-Phosphate holds strong potential for lung cancer treatment, though experimental validation is needed for clinical confirmation.

## 1. Introduction

Lung cancer remains one of the most lethal and complex malignancies globally, posing a significant public health challenge. It originates in the bronchial or alveolar epithelium and is marked by unchecked proliferation and a high propensity for early metastasis via lymphatic and hematogenous routes [1,2]. Often asymptomatic in early stages, lung cancer is typically diagnosed late, reducing treatment success and worsening prognosis. Rather than a single disease, it encompasses multiple histological and molecular subtypes with distinct behaviours and therapeutic responses [3,4]. According to the World Health Organisation (WHO) and the International Agency for Research on Cancer (IARC), lung cancer is the leading cause of cancer-related deaths worldwide. GLOBOCAN reported 2.21 million new cases and 1.8 million deaths in 2020 alone [5]. Projections indicate that mortality may surpass 3 million annually by 2050, driven by ageing populations, ongoing tobacco use, industrial and environmental carcinogen exposure, and disparities in healthcare access. This trend underscores the urgency for improved prevention, early detection, and therapeutic innovation. Lung cancer is broadly classified into non-small cell lung cancer (NSCLC, ~ 85%)—including adenocarcinoma, squamous cell carcinoma, and large cell carcinoma—and small cell lung cancer (SCLC, ~ 15%) [6–8]. NSCLC subtypes differ in location and smoking association, while SCLC is aggressive and initially chemo-sensitive but prone to resistant relapse. Rarer forms like carcinoid tumours and sarcomatoid carcinomas further highlight the disease’s heterogeneity. Tobacco smoking remains the primary risk factor, accounting for 85–90% of cases, due to carcinogens such as PAHs and nitrosamines [6,7]. However, a significant proportion—especially adenocarcinomas—arises in non-smokers, linked to secondhand smoke, air pollution (e.g., PM2.5), occupational exposures (e.g., radon, asbestos), and genetic predisposition. This multifactorial aetiology reinforces the need for precision medicine approaches tailored to individual tumour profiles [2].

Despite notable advancements in diagnostic imaging, histopathological classification, and molecular profiling, the clinical management of lung cancer remains fraught with challenges—chief among them the development of drug resistance. Drug resistance denotes the process by which cancer cells that initially respond to chemotherapeutic or targeted agents gradually acquire the ability to evade their cytotoxic effects, leading to therapeutic failure, disease progression, and relapse [9]. This resistance may be intrinsic (pre-existing within the tumour population) or acquired (emerging during treatment) and represents a formidable barrier to durable therapeutic success [10]. The underlying mechanisms are multifactorial and complex, encompassing genetic mutations, epigenetic reprogramming, phenotypic plasticity, increased expression of drug efflux pumps such as ATP-binding cassette (ABC) transporters, activation of alternative or compensatory signalling pathways, and modifications within the tumour microenvironment. For example, in NSCLC patients treated with EGFR-targeted tyrosine kinase inhibitors (TKIs), resistance frequently arises due to secondary mutations such as T790M, which alter drug-binding affinity. Tumours may also exploit bypass signalling routes—such as MET amplification or HER2 overexpression—to sustain proliferation despite upstream receptor blockade [11,12]. Moreover, processes like epithelial-to-mesenchymal transition (EMT), enrichment of cancer stem cell populations, and immune evasion further exacerbate resistance and reduce the durability of therapeutic response [13,14]. Given these challenges, multitargeted drug design has emerged as a transformative strategy in oncologic drug development. Unlike conventional monotherapies that act on single molecular targets, multitargeted therapies aim to concurrently disrupt several oncogenic proteins or signalling axes integral to tumour growth, survival, and metastatic potential [15–17]. This approach is informed by a systems biology framework, which views cancer not as a disease of isolated pathways but as a highly interconnected network of adaptive and redundant signalling circuits. Targeting multiple components within this network enhances therapeutic efficacy, mitigates escape risk via compensatory routes, and potentially delays or prevents resistance. Multitargeted drugs may take the form of single chemical entities with polypharmacological properties or rationally designed combination regimens involving agents with complementary mechanisms of action [11,18,19]. For instance, inhibitors simultaneously targeting EGFR and VEGFR pathways have demonstrated the ability to impair proliferative and angiogenic signalling, leading to more comprehensive tumour control. Importantly, such approaches may allow dose reductions of individual drugs, thereby minimising toxicity while maintaining efficacy. Multitargeted strategies also hold promise in addressing tumour heterogeneity—a hallmark of lung cancer—by engaging molecular drivers present across different subclonal populations [11,20]. This can result in more uniform and sustained responses, particularly in tumours with diverse mutational landscapes or those prone to clonal evolution under therapeutic pressure. Furthermore, these agents may disrupt critical feedback loops and crosstalk mechanisms that often underpin resistance to single-target therapies. Several multitargeted agents, such as cabozantinib (targeting MET, VEGFR, and AXL), crizotinib (targeting ALK, ROS1, and MET), and brigatinib (targeting ALK, EGFR, and ROS1), have already shown significant efficacy in NSCLC patients with refractory or resistant disease phenotypes [20].

Recent advancements in structural biology and bioinformatics have enabled the detailed characterisation of protein structures, facilitating rational drug design for complex diseases such as lung cancer [15,21,22]. Several proteins implicated in lung tumorigenesis have been structurally resolved and deposited in the Protein Data Bank (PDB), offering valuable targets for therapeutic intervention—particularly within multitargeted treatment strategies to overcome drug resistance. One such protein is Casein Kinase 2 (CK2; PDB ID: 1JWH), a serine/threonine kinase that regulates cell cycle progression, apoptosis inhibition, and transcription [23]. Overexpressed in lung cancer, CK2 modulates key oncogenic pathways through phosphorylation of substrates like PTEN, AKT, and p53. Its inhibition has been shown to reduce tumour viability and restore apoptosis, making it a compelling target in combination therapies designed to disrupt compensatory survival mechanisms [23]. The Ran-Importin β complex (PDB ID: 1IBR) governs nucleocytoplasmic transport, essential for maintaining transcriptional and cellular homeostasis [24]. In lung cancer, overexpression of Ran and Importin β contributes to oncogenesis by tumour suppressors (e.g., p53, FOXO) and promotes genomic instability [18]. Targeting this complex can restore proper protein localisation, enhance DNA damage responses, and sensitise cancer cells to chemotherapy when used in multitargeted regimens. Human Nerve Growth Factor (NGF; PDB ID: 1SG1), traditionally linked to neuronal development, also plays a role in tumour biology via its receptor TrkA [25]. NGF/TrkA signalling promotes cell proliferation, angiogenesis, and resistance to apoptosis through PI3K/AKT and MAPK/ERK pathways. In lung cancer, elevated NGF correlates with aggressive phenotypes and therapeutic resistance. Inhibiting NGF-TrkA interactions can suppress tumour progression and complement other inhibitors in multitargeted approaches. Together, these structurally characterised proteins—CK2, Ran-Importin β, and NGF—represent high-value targets in lung cancer therapy [23,24,25]. Their inhibition, particularly within the framework of multitargeted drug strategies, offers a promising avenue to dismantle the complex survival networks exploited by resistant tumour cells, ultimately enhancing treatment durability and clinical outcomes.

Human Survivin (PDB ID: 1XOX), a member of the inhibitor of apoptosis (IAP) family, plays dual roles in regulating cell division and inhibiting apoptosis [26]. While minimally expressed in normal adult tissues, survivin is markedly upregulated in lung cancers, where it supports tumour growth by blocking caspase activity and ensuring accurate mitotic spindle formation. Elevated survivin levels correlate with poor prognosis, enhanced proliferation, chemoradiotherapy resistance, and immune evasion. Its role in maintaining cancer stem cell populations further underscores its contribution to resistance. Structural insights from PDB ID: 1XOX reveal druggable pockets suitable for therapeutic targeting. Inhibiting survivin can restore apoptosis and disrupt mitosis, while its integration into multitargeted regimens enhances efficacy by eliminating resistant subclones and sensitising tumours to DNA-damaging agents or kinase inhibitors [26]. CRK-II (PDB ID: 2DVJ) is an adaptor protein central to signal transduction from growth factor receptors to downstream pathways controlling proliferation, adhesion, and migration [27]. In lung cancer, CRK-II is often overexpressed and drives epithelial-to-mesenchymal transition (EMT), metastasis, and resistance to tyrosine kinase inhibitors (TKIs). It mediates cytoskeletal reorganisation and enhances cell motility by interacting with c-Abl, FAK, and DOCK180. Inhibition of CRK-II disrupts these processes, curbing invasion and sensitising tumour cells to treatment. Multitargeted strategies, including CRK-II antagonism, provide a synergistic blockade of proliferative and metastatic tumour mechanisms [27]. Aldo-Keto Reductase Family 1 Member B10 (AKR1B10, (PDB ID: 4XZL), a cytosolic NADPH-dependent enzyme, is highly expressed in lung adenocarcinoma and squamous cell carcinoma [28]. AKR1B10 detoxifies reactive carbonyl species and supports lipid metabolism, aiding cancer cell survival under oxidative stress. It also modulates retinoic acid signalling and contributes to chemoresistance, particularly against agents that generate oxidative damage. Targeting AKR1B10 can compromise tumour redox homeostasis, reduce lipid synthesis, and potentiate chemotherapeutic response. Incorporating AKR1B10 inhibition in multitargeted therapies can disrupt metabolic and antioxidant pathways, weakening tumour defences and enhancing treatment efficacy [28]. Human tRNA dihydrouridine synthase 2 (hDus2), structurally represented by PDB ID: 4XP7, catalyses the reduction of uridine to dihydrouridine in tRNAs—an essential modification for RNA stability and translational efficiency [29]. Overexpressed in lung cancer, hDus2 supports tumour proliferation, survival, and resistance to apoptosis, particularly under cellular stress. Modulating protein synthesis contributes to cancer cells’ adaptive mechanisms to endure therapeutic pressure. Inhibiting hDus2 can impair translation and trigger apoptosis in rapidly dividing tumour cells. Thus, hDus2 represents a promising target within multitargeted lung cancer strategies [29]. Together, the seven proteins examined—CK2, Ran-Importin β, NGF, Survivin, CRK-II, AKR1B10, and hDus2—cover diverse but interrelated oncogenic functions, including signalling, transport, metabolism, and apoptosis regulation [23,24,25,28,29]. While individually targetable, the complexity and redundancy of tumour networks often render single-agent therapies ineffective. Multitargeted strategies offer a more robust therapeutic approach by inhibiting multiple critical pathways concurrently. Such combinations can overcome resistance, dismantle tumour adaptability, and improve clinical responses. By integrating inhibitors of CK2, NGF-TrkA, Survivin, and AKR1B10, future therapies can synergistically target cancer cell survival, proliferation, and metabolic flexibility, and characterisation of these targets enables the rational design of next-generation multitargeted therapeutics for lung cancer treatment [23,25,28].

In this study, our focus was on seven key proteins—CK2, Ran-Importin beta, NGF, Survivin, CRK-II, AKR1B10, and tRNA dihydrouridine synthase 2—due to their critical roles in lung cancer progression and therapeutic resistance. To identify a potent multitargeted candidate, we performed molecular docking of a curated DrugBank library to find compounds with strong binding across multiple targets. Molecular interaction fingerprints were generated to map interaction patterns between ligands and protein residues to elucidate the binding characteristics further. The top-performing compound was evaluated for drug-likeness and biological feasibility through pharmacokinetic profiling and DFT calculations. The thermodynamic and hydration behaviour of the ligand-bound complexes was assessed with 5 ns WaterMap simulations conducted for all seven protein-ligand systems. Additionally, we carried out 100 ns MD simulations to examine the complexes’ conformational stability and dynamic behaviour, and the resulting trajectories were employed to perform MM/GBSA binding free energy calculations for robust validation. The subsequent sections describe each methodological step with parameters.

## 2. Methods

**Fig 1:**
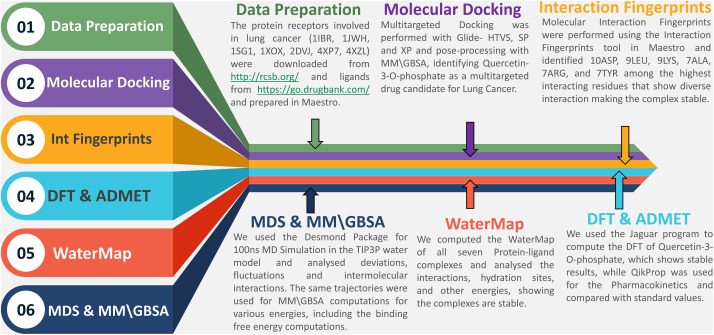
The workflow of the study shows the complete study from data collection to identification of the compound and then its validation using fingerprints, DFT, Pharmacokinetics, WaterMap, Molecular Dynamics and MM/GBSA-based validation of the complexes for energy level understanding.

The complete methods of the study have involved several steps, to make it easier to understand, and a workflow of the complete study is shown in Fig 1. Detailed methods are as follows-

### 2.1. Ligand library and structures preparation

A curated ligand library was developed comprising all compounds, including the FDA-approved drugs and small molecules, downloaded from the DrugBank database (https://go.drugbank.com/) [30]. The library was refined using LigPrep under OPLS4 force field constraints, with a molecular size filter of up to 500 atoms to eliminate excessively large or structurally ambiguous entities [31–33]. Ionisation states were predicted at physiological pH (7.0 ± 2.0) using Epik, with the application of desalt and tautomer generation steps [32,34,35]. A maximum of 32 stereoisomers per ligand was generated to account for stereochemical variations while preserving assigned chiral centres, and the final compound library was saved in SDF format and is ready for virtual screening workflows [32,33].

### 2.2. Obtaining protein’s crystal structure and its preparation

The foundation of any structure-based virtual screening workflow lies in the meticulous preparation of protein targets, as accurate receptor models are essential for reliable interaction predictions. In this study, we selected six distinct protein classes—transferase, cell cycle, growth factor, apoptosis, signalling, and oxidoreductase—each critically involved in lung cancer pathophysiology. Transferases regulate oncogenic pathways through abnormal group transfers. Cell cycle proteins drive uncontrolled proliferation by bypassing regulatory checkpoints. Growth factors stimulate angiogenesis and tumour expansion. Apoptotic proteins govern cell death pathways, often suppressed in cancer cells. Signalling proteins orchestrate downstream cascades influencing survival, invasion, and drug resistance. Oxidoreductases modulate redox balance, contributing to tumour progression and chemoresistance. Corresponding three-dimensional structures were retrieved from the RCSB Protein Data Bank (https://rcsb.org) with the following PDB IDs: 1JWH (transferase), 1IBR (cell cycle), 1SG1 (growth factor), 1XOX (apoptosis), 2DVJ (signalling), 4XZL and 4XP7 (oxidoreductase) [16,20,23,24,25–29,36]. These structures typically included multiple chains, bound cofactors, metal ions, and crystallographic waters. To ensure suitability for docking studies, each structure underwent processing using Schrödinger’s Protein Preparation Workflow within the Maestro (https://www.schrodinger.com/) interface [32,37]. The preparation pipeline comprised three major stages: (1) **Preprocessing**, which included capping terminal residues, assigning formal bond orders, forming disulfide bridges, and reconstructing any missing loops, side chains, or backbone atoms via the Prime module [32,38]. Metal coordination sites were maintained with zero bond orders. Protonation states of titratable residues were adjusted with Epik at physiological pH (7.4 ± 2) [32,35]. (2) **Optimisation**, where PROPKA was employed to fine-tune the protonation states of ionisable residues, orient water molecules and alleviate steric conflicts [32,39]. Hydrogen positions on titratable groups were optimised selectively. (3) **Energy Minimisation** was carried out using the OPLS4 force field until the heavy-atom RMSD reached a threshold of 0.30 Å [31,32]. Non-structural water molecules located within 5 Å of heteroatoms were deleted. For consistency, only the biologically active and structurally longest monomeric chain of each protein was retained for subsequent analyses.

### 2.3. Receptor grid generation and multitargeted docking strategies

For the screening of the ligand library against each protein and simulating the binding interactions, receptor grids were created around each target’s active site using the Receptor Grid Generation module in Maestro (https://www.schrodinger.com/) that defines the active site of a target protein where ligand binding is most likely to occur [32,40]. It creates a three-dimensional grid around the binding pocket, capturing the spatial and chemical environment. This step ensures that virtual screening or docking is focused only on biologically relevant regions, improving accuracy and efficiency in identifying potential drug candidates [12]. Grid box dimensions were tailored to include all active site residues, ensuring adequate space for ligand accommodation and flexibility for the proper docking calculations. Docking is a computational technique to predict how a small molecule (ligand) binds to a target protein’s active site. It evaluates various binding poses and estimates binding affinity using scoring functions. Docking plays a vital role in drug design by identifying lead compounds, optimising ligand orientation, and assessing molecular interactions, ultimately aiding in the rational development of effective and selective therapeutics. The prepared ligand set was screened using the Virtual Screening Workflow (VSW) tool backed by Glide (https://www.schrodinger.com/) [1,32,41–44]. Initially, pharmacokinetic parameters were assessed using QikProp and filters based on Lipinski’s Rule of Five to exclude compounds with poor drug-like properties [32,45,46]. Redundant entries were removed through molecular fingerprinting techniques. The docking strategy followed a multi-phase protocol: (1) High Throughput Virtual Screening (HTVS) to eliminate low-affinity compounds rapidly; (2) Standard Precision (SP) docking for further refinement of the top 50% hits from HTVS, and (3) Extra Precision (XP) docking for the top 10% of SP-scored compounds. Ionisation penalties were applied using Epik state penalties [32,35]. A van der Waals radius scaling factor of 0.80 and partial charge cutoff of 0.15 was set during docking to account for flexibility and electrostatics. The top-scoring XP-docked ligands (up to four poses per ligand) were subjected to post-docking evaluation using MM/GBSA (Molecular Mechanics Generalised Born Surface Area) calculations to estimate binding free energies. These energy values, docking scores, and poses were exported in CSV format for detailed downstream analysis. Furthermore, we have used the same grids with XP and MM/GBSA for the molecular docking with the Alectinib (DB11363), which is an FDA-approved drug molecule for lung cancer (control).

### 2.4. Molecular interaction fingerprints computations

Molecular Interaction Fingerprints (MIFs) were generated using the Maestro “Interaction Fingerprint” panel to capture and compare the molecular basis of protein-ligand interactions (https://www.schrodinger.com/). The top docked complexes involving Quercetin-3-O-phosphate with the four target proteins were analysed. All interaction types—including hydrogen bonds, π-π stacking, hydrophobic contacts, salt bridges, and others—were translated into binary fingerprint strings, allowing for systematic comparison [30,47–49]. The fingerprint data were aligned using PDB ID 1IBR as the structural reference. Non-interacting residues were excluded from the alignment to reduce noise, and visual mapping used a colour-coded scheme to differentiate N-terminal and C-terminal domains [32]. Mapping conserved interacting residues and docking affinities across the proteins enabled the identification of critical binding hotspots and highlighted Quercetin-3-O-phosphate’s multitarget potential.

### 2.5. Density Functional Theory and pharmacokinetics computations

Density Functional Theory (DFT) is a quantum mechanical approach used to study the electronic structure of drug molecules. DFT helps determine chemical reactivity, stability, and interaction potential in drug design with biological targets by analysing parameters such as HOMO-LUMO energies, electrostatic potentials, and molecular orbitals [32,50]. These insights aid in understanding how a drug binds to its target and optimises molecular properties for better efficacy. To evaluate the electronic properties and chemical reactivity of Quercetin-3-O-phosphate, Density Functional Theory (DFT) simulations were conducted using the Jaguar module in Maestro (https://www.schrodinger.com/) [32,51,52]. Geometry optimisations were performed using the B3LYP-D3 functional (including Grimme’s dispersion corrections) and the 6-21G** basis set [53]. The self-consistent field (SCF) method employed automatic spin configuration and atomic overlap for the initial guess [32,54]. Convergence criteria were set at 5 × 10 ⁻ ⁵ Hartree for energy changes and 5 × 10 ⁻ ⁶ for the RMS density matrix, with a cap of 48 SCF iterations [32,54]. The DIIS algorithm was used to accelerate convergence. Geometries were optimised using redundant internal coordinates and the Schlegel guess for the initial Hessian [32,55]. Optimisation was limited to a maximum of 100 steps under default convergence thresholds. Molecular descriptors such as electrostatic potential (ESP), average local ionisation energy, and non-covalent interaction fields were mapped on surfaces generated at 20 points/Å² density. Frontier molecular orbital energies (HOMO/LUMO) were calculated to evaluate the compound’s potential for electron donation or acceptance. The Poisson–Boltzmann Finite (PBF) model in water was used to simulate solvation, and free energies of solvation were derived from the gas-phase optimised structure. Energy progression was monitored using the QM-Monitor tool [32,51]. Pharmacokinetics describes how a drug is absorbed, distributed, metabolised, and excreted (ADME) in the body. In drug design, it helps assess a compound’s bioavailability, half-life, and toxicity risk. Evaluating pharmacokinetic profiles ensures that promising drug candidates are potent but also safe, effective, and capable of reaching their targets at therapeutic concentrations, thus increasing the likelihood of clinical success [56]. Additionally, pharmacokinetic profiling was carried out using QikProp, which provided key absorption, distribution, metabolism, and excretion (ADME) descriptors, and Lipinski’s Rule of Five was again applied to confirm drug-likeness [32,45,46,48].

### 2.6. WaterMap computations

WaterMap analysis assessed hydration’s thermodynamic contributions within each protein’s ligand-binding regions, and it identified energetically unfavourable water molecules that ligand atoms can displace to improve binding affinity and selectivity [41,57]. The docked complexes of Quercetin-3-O-phosphate with each protein target were subjected to WaterMap calculations in Maestro (https://www.schrodinger.com/). The system for each complex was trimmed to include residues within a 10 Å radius of the bound ligand. The OPLS4 force field was used, and crystallographic waters were retained to preserve native solvation environments [31,32]. Each system underwent a 5 ns molecular simulation (without trajectory generation) to capture hydration thermodynamics efficiently. Using the “Examine Results” panel, WaterMap visualised hydration sites, classifying them based on their enthalpic and entropic energy components. High-energy water molecules exhibiting positive ΔG were identified as favourable targets for displacement. Parameters such as overlap factor, hydration shell radius, and cumulative hydration site free energy were computed. Thermodynamic maps in 2D and 3D formats illustrated potential sites where ligand modifications could enhance binding via improved water displacement [41,57].

### 2.7. Molecular dynamics simulation

Molecular dynamics (MD) simulations provide insights into protein-ligand complexes’ temporal evolution and dynamic behaviour. This study performed all-atom MD simulations for each protein–Quercetin-3-O-phosphate complex using the Desmond engine within Maestro [32,58,59]. The System Builder module in Maestro (https://www.schrodinger.com/) was employed to solvate the complexes in an orthorhombic TIP3P water box, with a 10 Å buffer applied in all directions to ensure adequate hydration and avoid boundary artefacts [32,60]. Ionic neutralisation was achieved by adding counterions to offset the system charge; no additional salt was introduced, thereby preserving near-physiological ionic strength. Simulations were conducted under an NPT (constant number of particles, pressure, and temperature) ensemble for 100 nanoseconds (ns), sufficient to observe binding stability and structural fluctuations [32,61]. Temperature was maintained at 300 K using the Nosé–Hoover thermostat, and pressure was set at 1.01325 bar (1 atm) using the Martyna–Tuckerman–Klein barostat—standard biological conditions. Trajectories were recorded every 100 picoseconds (ps), yielding 1000 frames for robust post-simulation analysis. Preceding the production run, energy minimisation and relaxation protocols ensured the elimination of steric clashes and the attainment of system equilibrium. Key dynamic descriptors—root mean square deviation (RMSD), root mean square fluctuation (RMSF), and interaction timelines—were analysed using the Simulation Interaction Diagram (SID) module (https://www.schrodinger.com/) [32,58]. These metrics helped assess complex stability, conformational flexibility, and the persistence of critical intermolecular contacts.

### 2.8. Binding free energy computations

MM/GBSA (Molecular Mechanics/Generalised Born Surface Area) on MD simulation trajectories estimates the binding free energy of protein-ligand complexes over time. It combines molecular mechanics energies with solvation effects and is applied to multiple frames from the MD trajectory to account for conformational flexibility. This approach offers a more realistic and averaged assessment of binding affinity, making it essential for evaluating the stability and strength of interactions in dynamic biological environments. The binding free energies, total complex energies, and many other energies were calculated using the MM/GBSA approach [62]. A custom Python script (thermal_mmgbsa.py) was run from the Ubuntu terminal using the command:

*$SCHRODINGER/run thermal_mmgbsa.py JOB_NAME-out.cms*

The script computed ΔG_bind values for all 1000 frames of each trajectory. The total binding free energy components included van der Waals interactions, electrostatics, polar solvation, and nonpolar solvation contributions. Time-resolved energy plots enabled the evaluation of convergence and thermodynamic consistency. Together with MD and docking outputs, these calculations validated ligand stability and binding favourability under near-physiological conditions [20,62,63].

## 3. Results

In this comprehensive study, we aimed to identify a multitargeted drug compound using multitarget molecular docking, and we identified Quercetin-3-O-phosphate as a potential drug candidate, validating its efficacy computationally. Further, detailed results for each step are as follows.

### 3.1. Analysis and evaluation of the prepared protein structures

**Fig 2:**
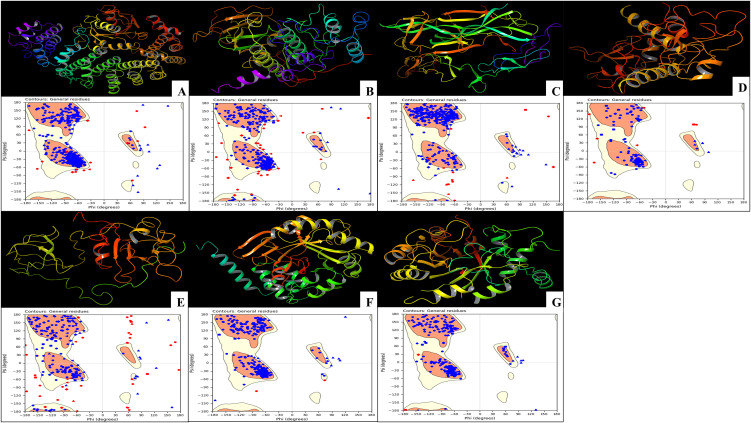
Prepared Protein Structures and Ramachandran plot for A) CK2 kinase (PDB ID: 1JWH), B) Ran-importin β (PDB ID: 1IBR), C) Human nerve growth factor (PDB ID: 1SG1), D) Human survivin structure (PDB ID: 1XOX), E) CRK-II (PDB ID: 2DVJ), F) tRNA dihydrouridine synthase 2 (PDB ID: 4XP7), and G) Human AKR1B10 (PDB ID: 4XZL).

The analysis of protein energies generated through the Protein Preparation Workflow in Schrödinger Maestro provides critical insights into the stability and quality of the molecular systems before simulation or docking studies. The energies evaluated encompass various physical interactions, collectively defining each protein structure’s conformational viability and thermodynamic favourability. These energy components, particularly the total and potential energies, serve as indicative metrics for the overall stability of the protein in its optimised form. All structures reported here show zero kinetic energy and temperature, reflecting their energy-minimised, static state rather than any dynamics-based simulations. PDB ID 1IBR retains the most favourable energy profile with a total and potential energy of approximately −6290 kcal/mol. This highly stabilised state results from significant contributions of non-bonded interactions, particularly Lennard-Jones energy at −12687.4 kcal/mol and electrostatic energy at −7395.96 kcal/mol. These strong, attractive forces are balanced by considerable internal strain, as evident from bond stretch (602.65 kcal/mol), angle bending (2773.68 kcal/mol), and torsional (2473.3 kcal/mol) energies. The positive values in these bonded terms reflect the energetic cost of maintaining the protein’s covalent geometry and backbone flexibility, which are offset by dominant non-bonded attractions, producing a highly stable configuration. PDB ID 1JWH follows with a total energy of −3090 kcal/mol, which, while still thermodynamically favourable, is notably less stable than 1IBR. This is consistent with the slightly lower Lennard-Jones (−10245.8 kcal/mol) and electrostatic (−5625.61 kcal/mol) energies, indicating weaker non-bonded interactions. The internal strain remains substantial with bond stretching at 495.76 kcal/mol and high values in angle bending (2552.96 kcal/mol) and torsion angles (2306.55 kcal/mol). These findings point to a structure that may be well-folded but under strain, possibly due to tight packing or constrained loop regions. For PDB ID 1SG1, the total energy increases (less negative) to −2150 kcal/mol, suggesting moderate stabilisation. The bond stretch energy (155.76 kcal/mol), angle bending (691.35 kcal/mol), and torsion energy (774.7 kcal/mol) indicate lower internal strain than in 1IBR or 1JWH. While these reduced strain values are energetically favourable, the corresponding drop in stabilising Lennard-Jones (−3479.58 kcal/mol) and electrostatic (−2750.12 kcal/mol) energies results in less negative total energy. This implies a more relaxed, possibly more flexible structure, but with reduced compactness and fewer stabilising interactions. PDB ID 1XOX shows a significant jump in total energy to −134 kcal/mol, making it a less stabilised structure, albeit still negative. Both bonded and non-bonded energy terms are modest: bond stretch is 117.57 kcal/mol, angle bending is 577.04 kcal/mol, and torsion is 599.85 kcal/mol, with Lennard-Jones and electrostatics at −2011.73 kcal/mol and −1104.32 kcal/mol, respectively. These lower magnitudes suggest weaker interatomic contacts and minimal internal strain, indicative of a loosely packed and possibly partially unfolded or structurally less optimised conformation. Notably, the value for PDB ID 2DVJ shows a total energy of −473.13 kcal/mol, revising the previous interpretation of instability. Despite being the second least negative total energy, it is now within a thermodynamically permissible range. The internal strain is modest, with bond stretch (106.12 kcal/mol), angle bending (602.63 kcal/mol), and torsion (734.72 kcal/mol) energies, while non-bonded stabilisation is limited, as reflected by Lennard-Jones (−1590.22 kcal/mol) and electrostatic (−865.07 kcal/mol) interactions. Although this structure is less stabilised than others, it no longer appears energetically pathological and may represent a flexible or partially solvent-exposed conformation. PDB IDs 4XP7 and 4XZL display similar profiles, with total energies of −2399.4 kcal/mol and −2208.22 kcal/mol, respectively. Both structures maintain a balance between moderate bonded strain (bond stretch energies of ~161.99 and 170.92 kcal/mol; angle bending ~708.89 and 754.58 kcal/mol) and significant non-bonded interactions (Lennard-Jones ~ −3530.51 and −3600.84 kcal/mol; electrostatic ~ −2240.67 and −2311.42 kcal/mol). The relatively even distribution of strain and stabilisation energies supports the conclusion that these proteins are in well-prepared, stable conformations with compact and energetically favourable folds. The energy profiles confirm that all seven protein structures fall within acceptable thermodynamic ranges post-preparation, with varying degrees of internal strain and non-bonded stabilisation. Minor variation is not inherently problematic but should be considered in downstream applications, particularly in docking or dynamic simulations, where conformational stability is paramount. Furthermore, Fig 2 has prepared 3D protein structures, ligand binding sites and their Ramachandran Plots to understand everything properly.

### 3.2. Analysis of interactions and energies from multitargeted docking

**Fig 3:**
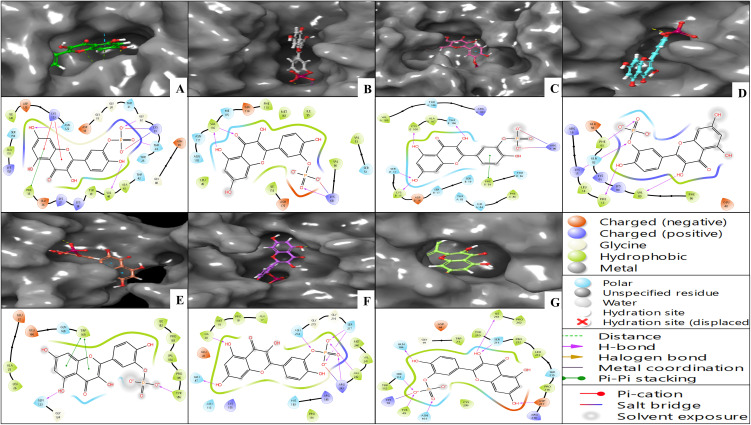
3D and 2D ligand Interaction Diagram of structures of Quercetin-3-O-phosphate in complex with A) CK2 kinase (PDB ID: 1JWH), B) Ran-importin β (PDB ID: 1IBR), C) Human nerve growth factor (PDB ID: 1SG1), D) Human survivin structure (PDB ID: 1XOX), E) CRK-II (PDB ID: 2DVJ), F) tRNA dihydrouridine synthase 2 (PDB ID: 4XP7), and G) Human AKR1B10 (PDB ID: 4XZL).

The multitargeted molecular docking study has revealed several potential candidates, but Quercetin-3-O-phosphate has emerged as the top candidate for all seven protein targets, which were conducted using a precisely defined grid-based docking protocol in Schrödinger Maestro, followed by MM/GBSA free energy calculations to refine binding energetics and elucidate the quality of interactions at the molecular level. Grid generation involved the placement of grid boxes centred on each receptor’s predicted or known active sites, with dimensions tailored to encompass critical binding pockets based on crystallographic information or homologous modelling. The grid centre coordinates were precisely defined to guide ligand sampling and scoring accurately. In this context, the stretching energy (S-OPLS) obtained during system minimisation was also considered to assess local strain within the protein binding pocket and its potential influence on ligand accommodation [31,32]. For the CK2 kinase (PDB ID: 1JWH), which has a resolution of 3.1 Å, the docking grid was centred at (0.631, 56.926, −54.579), positioning the search space accurately around the active site. The docking score was recorded at −11.63 kcal/mol, supported by an XP GScore of −11.73 kcal/mol and a ligand efficiency of −1.093 kcal/mol per heavy atom, indicating a strong and specific binding affinity. MM/GBSA analysis yielded a ΔG_bind of −18.95 kcal/mol. Key interactions include three hydrogen bonds involving VAL116 and LYS68, along with the formation of a salt bridge between LYS68 and the phosphate group of Quercetin-3-O-phosphate. This binding mode is consistent with moderate local stretch energy (368.792 kcal/mol), and the overall complex energy was −13,563.11 kcal/mol, reflecting a stable interaction (Table 1, Fig 3). In the case of Ran-importin β (PDB ID: 1IBR), with a resolution of 2.3 Å and a grid centre at (−6.564, −5.193, 28.267), docking produced a score of −10.10 kcal/mol, with an XP GScore of −10.2 kcal/mol and a notably favourable ligand efficiency of −2.716 kcal/mol. MM/GBSA binding free energy was highly favourable at −47.07 kcal/mol, further supported by a hydrogen bond-specific MM/GBSA contribution of −6.97 kcal/mol. This complex exhibited the strongest MM/GBSA score across all tested proteins. The protein-ligand complex engaged multiple hydrogen bonds, particularly with residues VAL40, ASN122, GLY22, and THR24, and included π-cation interactions with LYS123 and π-π stacking with PHE35. Salt bridges were observed between the ligand and LYS23. The S-OPLS stretch energy was relatively high at 563.584 kcal/mol, likely reflective of conformational tension in a highly interactive binding pocket, yet the complex energy of −25,786.02 kcal/mol demonstrates a deeply stabilised protein-ligand ensemble (Table 1, Fig 3). The Human nerve growth factor (PDB ID: 1SG1), resolved at 2.4 Å and with a grid centred at (−0.523, 60.469, −20.127), yielded a docking score of −8.14 kcal/mol and a less favourable ligand efficiency of −0.824 kcal/mol. The XP hydrogen bond contribution was substantial (−4.268 kcal/mol), consistent with the presence of four hydrogen bonds involving SER13, CYS15, THR106, and CYS108, with MM/GBSA ΔG_bind calculated at −14.28 kcal/mol. A π-π stacking interaction was observed with PHE54, while ARG80 contributed two salt bridges with different phosphate oxygen atoms. The moderate S-OPLS stretch energy (142.926 kcal/mol) indicates a relatively relaxed active site. The total complex energy of −14,352.29 kcal/mol reflects overall system stability (Table 1, Fig 3). In the Human survivin structure (PDB ID: 1XOX)—which lacks resolved crystallographic data (resolution NA)—a grid was placed at (−25.571, −47.629, −83.623), suggesting a modelled or inferred active site. The docking score was −5.84 kcal/mol, with a ligand efficiency of −1.103 kcal/mol and MM/GBSA ΔG_bind of −19.12 kcal/mol. The interactions involved hydrogen bonding with PHE13, VAL89, LYS15, and PHE93. Despite relatively weaker scoring terms and the lowest complex energy (−4572.28 kcal/mol) among all structures, the binding was reinforced by a moderate stretch energy of 111.941 kcal/mol, indicating a somewhat strained but viable binding environment (Table 1, Fig 3). The CRK-II (PDB ID: 2DVJ), also lacking crystallographic resolution data, used a grid centred at (347.392, 1.552, −12.256), targeting a presumed allosteric or active site. The docking score of −6.00 kcal/mol and the MM/GBSA score of −22.12 kcal/mol place this protein in a moderate binding category. Hydrogen bonding interactions were observed between GLN123 and TYR186 with the ligand’s OH and phosphate oxygen atoms, respectively. Two π-π stacking interactions were noted involving TRP169. The relatively low S-OPLS stretch energy (83.045 kcal/mol) and complex energy of −7279.97 kcal/mol suggest a stable yet flexible interaction landscape (Table 1, Fig 3). In the docking of tRNA dihydrouridine synthase 2 (PDB ID: 4XP7), with a high-resolution structure at 1.9 Å, the grid centred at (10.893, 7.13, 14.378) revealed a high-affinity binding site with a docking score of −11.15 kcal/mol and MM/GBSA binding free energy of −37.12 kcal/mol. Extensive hydrogen bonding was observed with residues VAL20 and GLN87 via two OH groups, and four O atoms formed hydrogen bonds with ASN214, SER217, ALA242, and ARG243. A salt bridge was formed with ARG243, further enhancing stability. These results correlate with an S-OPLS stretch energy of 158.672 kcal/mol and a complex energy of −14,179.98 kcal/mol, reflecting strong structural complementarity and energetically favourable interactions (Table 1, Fig 3). Lastly, Human AKR1B10 (PDB ID: 4XZL), with the highest crystallographic resolution among the targets (1.7 Å), showed a docking score of −9.54 kcal/mol, an XP GScore of −9.64 kcal/mol, and MM/GBSA ΔG_bind of −32.47 kcal/mol. The binding site (−10.79, 25.027, −0.561) accommodated the ligand with a favourable ligand efficiency of −1.873 kcal/mol. Hydrogen bonding involved GLN184, SER211, ASP217, and ILE261 via OH atoms and TYR49, HIE111, and ASN161 via O atoms. A salt bridge interaction with LYS78 was also recorded. The stretch energy was 153.295 kcal/mol, and the complex energy was −10,383.21 kcal/mol, indicative of a robust and well-defined binding interface (Table 1, Fig 3). The integration of grid-based docking, energy minimisation parameters such as S-OPLS stretch energy, and post-docking MM/GBSA calculations reveals that Quercetin-3-O-phosphate engages in a strong and specific binding with several proteins, particularly 1IBR and 4XP7. These interactions are characterised by rich non-covalent interactions—hydrogen bonds, salt bridges, and π-stacking—supported by favourable docking scores and complex energies. Such data provide a solid structural and energetic rationale for selecting promising protein targets for further pharmacological or computational studies. Moreover, we compared the results of Quercetin-3-O-Phosphate with the FDA-approved drug Alectinib (DB11363), where Quercetin-3-O-Phosphate showed superior performance (see Supplementary File (doc) and Supplementary Sheets). Therefore, Quercetin-3-O-Phosphate is strongly recommended for further investigation.

**Table 1:** Showing the protein information, docking and other energies (kcal/mol) produced during the multitargeted molecular docking studies (against Quercetin-3-O-phosphate).

PDB ID	Resolution	gridbox xcent	gridbox ycent	gridbox zcent	Stch Energy-S-OPLS	Docking Score	XP GScore
1IBR	2.3	−6.564	−5.193	28.267	563.584	−10.099	−10.2
1JWH	3.1	0.631	56.926	−54.579	368.792	−11.627	−11.729
1SG1	2.4	−0.523	60.469	−20.127	142.926	−8.143	−8.244
1XOX	NA	−25.571	−47.629	−83.623	111.941	−5.835	−5.936
2DVJ	NA	347.392	1.552	−12.256	83.045	−6.009	−6.11
4XP7	1.9	10.893	7.13	14.378	158.672	−11.153	−11.254
4XZL	1.7	−10.79	25.027	−0.561	153.295	−9.535	−9.637
PDB ID	**ligand efficiency sa**	**ligand efficiency In**	**Complex Energy**	**XP HBond**	**MM/GBSA dG Bind**	**MM/GBSA dG Bind Hbond**	**Receptor Hbond**
1IBR	−2.716	−11.055	−25786.018	−2.865	−47.07	−6.97	−358.842
1JWH	−1.093	−4.449	−13563.108	−1.677	−18.95	−2.94	−174.276
1SG1	−0.824	−3.355	−14352.291	−4.268	−14.28	−5.39	−151.043
1XOX	−1.103	−4.49	−4572.28	−2.783	−19.12	−2.41	−38.51
2DVJ	−1.276	−5.195	−7279.972	−2.084	−22.12	−1.35	−60.145
4XP7	−2.142	−8.718	−14179.976	−3.989	−37.12	−8.68	−188.719
4XZL	−1.873	−7.625	−10383.207	−3.063	−32.47	−4.91	−138.287

### 3.3. Analysis of molecular interaction fingerprints

**Fig 4:**
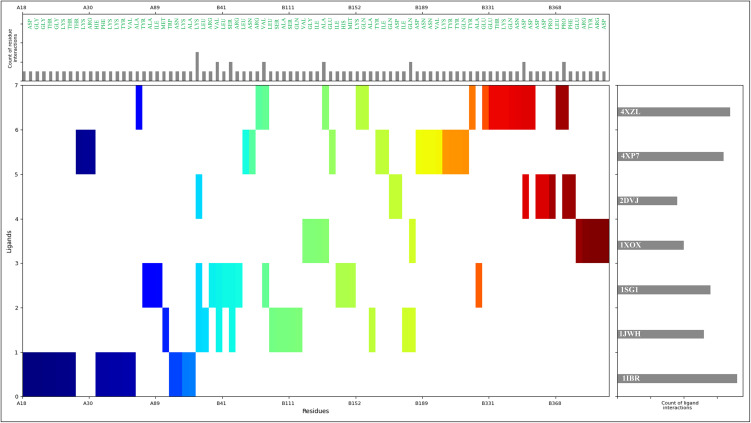
Showing the Molecular Interaction Fingerprints computed on the docked poses of Quercetin-3-O-phosphate in complex with A) CK2 kinase (PDB ID: 1JWH), B) Ran-importin β (PDB ID: 1IBR), C) Human nerve growth factor (PDB ID: 1SG1), D) Human survivin structure (PDB ID: 1XOX), E) CRK-II (PDB ID: 2DVJ), F) tRNA dihydrouridine synthase 2 (PDB ID: 4XP7), and G) Human AKR1B10 (PDB ID: 4XZL).

The interaction fingerprints generated for the ligand Quercetin-3-O-phosphate against the protein targets revealed a detailed picture of the specific residues involved in forming the protein-ligand complex. The list of residues and their frequencies offers insight into the predominant types of interactions between the ligand and the protein, as well as the characteristics of the ligand binding pocket. Aspartic acid (ASP) is the most frequently involved residue, comprising 10 occurrences. This polar, negatively charged residue is known for its ability to form strong electrostatic interactions, particularly hydrogen bonds with positively charged groups or polar ligands. In this case, the high number of interactions between ASP and the ligand suggests the involvement of ionic interactions or hydrogen bonds, particularly with the hydroxyl and phosphate groups on Quercetin-3-O-phosphate. The phosphate group, being negatively charged, would likely form a stable salt bridge or hydrogen bond with the negatively charged carboxyl group of ASP, enhancing the overall electrostatic complementarity and stability of the complex. Leucine (LEU) and Lysine (LYS) are tied for second place, appearing 9 times (Fig 4). LEU, a hydrophobic amino acid, is involved in nonpolar interactions that help stabilise the complex through van der Waals forces and hydrophobic packing. Numerous LEU residues reflect a strong hydrophobic interaction between the ligand and the protein, which could be attributed to the aromatic benzene rings of Quercetin-3-O-phosphate. These interactions likely provide a stable core to the protein-ligand interface, contributing to the overall structural rigidity of the complex. On the other hand, LYS, a positively charged residue, plays an important role in forming salt bridges and hydrogen bonds with the ligand, particularly with the phosphate group, further enhancing the electrostatic complementarity of the complex. The significant presence of LYS suggests that Quercetin-3-O-phosphate might preferentially bind to regions where both hydrophobic and electrostatic interactions are balanced, providing a stable interface. Alanine (ALA), Arginine (ARG), and Tyrosine (TYR) all appear 7 times in the interaction fingerprint, and their involvement reflects a diverse set of interactions that contribute to the ligand’s binding affinity. ALA, being a small, nonpolar residue, likely participates in hydrophobic interactions with the ligand, helping to stabilise the nonpolar regions of the molecule, particularly the aromatic rings. ARG, a positively charged, polar residue, suggests that Quercetin-3-O-phosphate interacts with the ligand through electrostatic attraction, especially with the negatively charged phosphate group. Additionally, TYR, a polar amino acid with a phenolic group, can engage in both hydrogen bonding and hydrophobic interactions with the ligand. The aromatic nature of TYR can contribute to π-π stacking interactions, particularly with the benzene rings in Quercetin-3-O-phosphate, further stabilising the complex through aromatic interactions. The residues Glutamine (GLN) and Asparagine (ASN), which appear 6 and 5 times, respectively, are polar and hydrophilic amino acids capable of forming hydrogen bonds with the hydroxyl and phosphate groups of Quercetin-3-O-phosphate (Fig 4). These residues help solvate the ligand and stabilise the protein-ligand complex through polar interactions, especially in protein regions where hydration and proper orientation of functional groups are crucial for maintaining complex stability. Glutamic acid (GLU) and Isoleucine (ILE), which appear 5 times each, contribute significantly to the ligand’s binding stability. Similar to ASP, GLU is negatively charged and can form ionic bonds or hydrogen bonds with positively charged groups or polar residues on the ligand. This type of interaction contributes to the overall electrostatic stabilisation of the complex. ILE, a hydrophobic residue, is likely involved in nonpolar interactions, similar to LEU, further supporting the hydrophobic core of the binding site. Valine (VAL), with 5 occurrences, is a branched, nonpolar amino acid that contributes to hydrophobic interactions within the binding pocket, providing further stabilisation to the complex. Its role is similar to that of LEU and ALA in contributing to the hydrophobic environment and aiding in the packing of the protein-ligand interface. Glycine (GLY) and Threonine (THR), appearing 4 times each, contribute differently. GLY, the smallest amino acid, likely helps to maintain flexibility within the binding pocket, allowing for proper ligand accommodation and enhancing the binding site’s adaptability. THR, a polar residue with a hydroxyl group, can form hydrogen bonds with the hydroxyl and phosphate groups of Quercetin-3-O-phosphate, stabilising the ligand in the protein binding pocket. The occurrence of Proline (PRO) and Serine (SER), both at 3 times each (Fig 4), suggests that these residues might be involved in maintaining the structural integrity of the protein during ligand binding. PRO’s unique cyclic structure often induces rigidity in protein structures, potentially helping to stabilise the binding site. With its hydroxyl group, SER can bind hydrogen with the ligand, further stabilising the interaction. Histidine (HIS) and Methionine (MET), with 2 occurrences each, are less involved but still play a role in the overall stability of the complex. HIS, a polar residue with an imidazole group, can participate in hydrogen bonding and potentially in metal coordination if present, while MET, with its sulphur-containing side chain, might contribute to hydrophobic and van der Waals interactions. Finally, Phenylalanine (PHE), which appears twice, is another aromatic residue that can contribute to π-π stacking interactions with the benzene rings of Quercetin-3-O-phosphate, further enhancing the aromatic interactions within the protein-ligand complex. A single tryptophan (TRP) residue, which is highly hydrophobic and capable of engaging in π-π stacking interactions, also supports the aromaticity and hydrophobic nature of the binding site. The residue interaction pattern suggests that Quercetin-3-O-phosphate binds predominantly through a combination of hydrophobic, electrostatic, and hydrogen bonding interactions, with a notable preference for residues such as ASP, LYS, and LEU. These residues contribute to a favourable binding pocket characterised by hydrophobic and electrostatic interactions, indicating that the ligand prefers binding to well-organised pockets to accommodate both polar and nonpolar interactions. The predominance of hydrophobic residues such as LEU, ALA, TYR, and ILE indicates that Quercetin-3-O-phosphate might be primarily interacting through its aromatic and nonpolar regions, with its phosphate and hydroxyl groups forming critical polar interactions that further stabilise the complex. This comprehensive residue interaction profile highlights the importance of a balanced and synergistic network of interactions, crucial for the effective binding of Quercetin-3-O-phosphate to the protein targets.

### 3.4. Analysis of density functional theory

**Fig 5:**
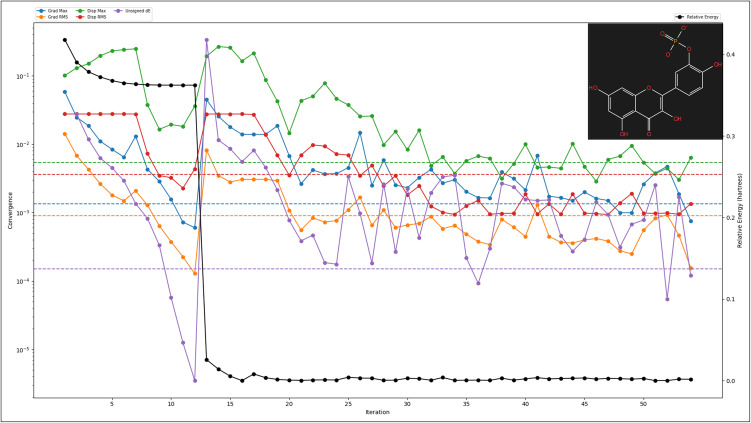
Showing the Density Functional Theory (DFT) results of Quercetin-3-O-phosphate computed using Jaguar.

The DFT (Density Functional Theory) results computed for Quercetin-3-O-Phosphate using the B3LYP-D3 functional in the Jaguar module provide a detailed electronic and thermodynamic description of the molecule, offering insights into its stability, electronic structure, and solvation characteristics. The spin multiplicity of the system is reported as 1, indicating that Quercetin-3-O-Phosphate is in its singlet ground state, which is typical for most neutral organic molecules. The QM method used, DFT(B3LYP-D3)/SOLV, is a hybrid functional that incorporates dispersion corrections (D3) for better modelling of non-covalent interactions, and it also includes solvent effects via a solvation model. This allows for a more accurate description of the molecule’s behaviour in solution, which is critical for understanding its interactions in a biological context. The geometry convergence category of 4 indicates that the optimisation process has successfully converged to a local minimum on the potential energy surface, ensuring that the final structure is stable and that no significant energy is required to move the system towards equilibrium. This result suggests that the optimised geometry of Quercetin-3-O-Phosphate is energetically favourable, providing a reliable foundation for further analysis. In the gas phase, the computed energy is −1670.722362 Hartrees, and in the solution phase, the energy is slightly lower at −1671.082947 Hartrees, indicating that the molecule’s interaction with the solvent leads to a favourable stabilisation. The solvation energy of −226.27 kcal/mol reveals the extent to which the solvent stabilises the molecule, which is substantial and suggests that solvation plays a key role in the stability of the ligand in a biological environment. The HOMO (Highest Occupied Molecular Orbital) and LUMO (Lowest Unoccupied Molecular Orbital) energies are −0.20563 eV and −0.068237 eV, respectively (Fig 5). The small energy gap between the HOMO and LUMO reflects the molecule’s potential for electron transfer and suggests that Quercetin-3-O-Phosphate might be reactive or capable of participating in charge transfer processes, which could be important for its biological activity. This small gap also indicates that the molecule is electronically flexible, typical of many natural compounds with bioactivity, allowing it to interact with various biological targets. The lowest frequency for Quercetin-3-O-Phosphate is 59.941 cm-1, a very low vibrational mode that typically corresponds to large-amplitude motions, such as those associated with molecular rotations or translations, and suggests that the molecule has a low-lying vibrational mode that could be important for flexibility. The highest frequency of 3701.437 cm-1 represents the stretching modes of bonds, typically associated with the higher energy vibrational modes of the molecule, likely corresponding to bond stretching in the aromatic rings and the phosphate group. These vibrational frequencies provide insight into the molecular motions and stability of the structure. The zero-point energy (ZPE) of 146.805 kcal/mol corresponds to the energy in the system at absolute zero temperature due to quantum mechanical effects (Fig 5). This is an important thermodynamic quantity, indicating the amount of energy that must be supplied to the molecule to reach its ground state. The entropy at 298.15K is 143.959 cal/mol/K, reflecting the degree of disorder in the system. This relatively high entropy suggests that Quercetin-3-O-Phosphate, a flexible molecule, may exhibit significant conformational variability, contributing to its biological activity by enabling various binding modes or interactions with target proteins. The enthalpy at 298.15K is 13.428824 kcal/mol, which is the total heat content of the molecule at this temperature, while the free energy of −29.492639 kcal/mol represents the Gibbs free energy at standard conditions (Fig 5). The negative value of the free energy indicates that the optimised structure of Quercetin-3-O-Phosphate is thermodynamically favourable, with the molecule being stable in the solution phase. The internal energy of the molecule at 298.15K is 12.836338 kcal/mol, which, when combined with the free energy and enthalpy, helps to characterise the system’s stability and the distribution of energy within the molecule. The heat capacity at 298.15K is 83.14 cal/mol/K, which measures the molecule’s ability to absorb heat and indicates the extent of molecular vibrations. The ALIE (Atomic Localized Ionization Energy) is reported for Quercetin-3-O-Phosphate with the following statistics: the min ALIE is 183.99 kcal/mol, the max ALIE is 377.1 kcal/mol, and the mean ALIE is 255.27 kcal/mol. ALIE measures the energy required to ionise atoms in the molecule, providing insight into the relative reactivity of different molecule regions (Fig 5). The relatively high range of ALIE values suggests that some regions of Quercetin-3-O-Phosphate are more reactive than others and that the molecule might have regions of localised electron density prone to interaction with electrophilic or nucleophilic species. The variance in ALIE values is 1596.19 kcal/mol², indicating significant variability in the ionisation energies of the atoms across the molecule, further supporting the notion of a molecule with varying reactivity. The ALIE balance of 0 suggests that the ionisation energy distribution is evenly spread across the molecule, which might indicate that Quercetin-3-O-Phosphate is relatively stable overall but with localised regions that are more reactive. The average absolute deviation from the mean ALIE is 32.859403 kcal/mol, a measure of the distribution spread and suggesting that the variation in reactivity across the molecule is relatively moderate. This, combined with the high ALIE values, implies that Quercetin-3-O-Phosphate may exhibit selective reactivity depending on its chemical environment. The DFT results reveal that Quercetin-3-O-Phosphate is a stable molecule in gas and solution phases, with significant solvation energy stabilising its conformation in a biological environment. The electronic structure, indicated by the HOMO-LUMO gap, and the thermodynamic properties suggest that the molecule is thermodynamically stable and potentially reactive under certain conditions, making it an excellent candidate for further studies on its interactions and potential biological activity. The ALIE values and entropy further support the flexibility and reactivity of the molecule, which is essential for its interactions with biological macromolecules like proteins.

### 3.5. Analysis of pharmacokinetics

The pharmacokinetic properties of Quercetin-3-O-Phosphate, as computed using the QikProp module in Schrödinger. The molecular volume of Quercetin-3-O-Phosphate is 1023.313 Å³, which falls within the typical range of small drug-like molecules (500–2000 Å³). This suggests the ligand is reasonably sized for passive diffusion across cell membranes. The molecular weight (MW) is not explicitly provided, but based on the volume, the molecule seems to fit within the desired parameters for drug-like candidates, generally between 130 and 725 Da. Regarding polar surface area (PSA), Quercetin-3-O-Phosphate has a value of 189.97 Å², at the higher end of the recommended range for drug candidates (7.0–200.0 Å²) (Table 2). A high PSA often correlates with enhanced solubility and good interaction with water-soluble biological systems, but can also hinder membrane permeability if excessively large. The SASA (Solvent Accessible Surface Area) of 601.406 Å² further supports the solubility properties of Quercetin-3-O-Phosphate, placing it within the typical range for small molecules that need to interact with water-soluble components, such as proteins or other biomolecules in the bloodstream. The LogP values, which describe the molecule’s hydrophobicity, are important for understanding its ability to cross biological membranes. QPlogP is calculated to be 20.144, which is relatively high, indicating a strong hydrophobic character, while QPlogPoct (logarithm of the partition coefficient between octanol and water) is 25.8, which further emphasises the molecule’s lipophilic nature. These high values suggest that Quercetin-3-O-Phosphate will likely be more distributed in the lipid phase, indicating potential difficulties in aqueous solubility or membrane permeability. However, this could be compensated for by the presence of the phosphate group, which can increase solubility in aqueous media. The human oral absorption value for Quercetin-3-O-Phosphate is calculated at 9.5%, which is considered low. This suggests that Quercetin-3-O-Phosphate may face challenges in being absorbed efficiently from the gastrointestinal tract if administered orally. This relatively low oral absorption could be due to its larger size, higher polarity, and possibly its limited solubility, all of which are common characteristics of molecules with poor bioavailability. For the blood-brain barrier (BBB) penetration, QPlogBB is reported as −3.874, which is significantly lower than the ideal threshold of −1.0 or above, suggesting that Quercetin-3-O-Phosphate will not readily cross the blood-brain barrier (Table 2). This is typical for highly charged or large molecules and may restrict their potential neuropharmacological applications. The Caco-2 cell permeability (QPPCaco) is calculated at 0.11 × 10 ⁻ ⁶ cm/s, which indicates poor permeability across the intestinal barrier. This reinforces the notion of limited oral absorption, suggesting that the molecule may face challenges in reaching effective concentrations in systemic circulation through oral administration. The logS (solubility) of Quercetin-3-O-Phosphate is calculated at −2.88, which falls within the range for molecules poorly soluble in water (the ideal range is between −6.5 and 0.5). This result further supports the difficulty in the oral absorption of Quercetin-3-O-Phosphate, as solubility is a

**Table 2:** Pharmacokinetics Results of Quercetin-3-O-phosphate were computed using the QikProp and compared with its standard values.

Descriptors	Standard Values	Quercetin-3-O-Phosphate	Descriptors	Standard Values	Quercetin-3-O-Phosphate
WPSA	0.0–175.0	4.554	glob	0.75–0.95	1
volume	500.0–2000.0	1023.313	FOSA	0.0–750.0	0.8165876
Type	N/A	small	FISA	7.0–330.0	0
SASA	300.0–1000.0	601.406	EA(eV)	−0.9–1.7	396.707
SAfluorine	0.0–100.0	0	donorHB	0.0–6.0	0.57
SAamideO	0.0–35.0	0	dipole	1.0–12.5	5
RuleOfThree	maximum is 3	1	dip^2/V	0.0–0.13	12.485
RuleOfFive	maximum is 4	1	CNS	−2 (inactive), + 2 (active)	0.1523162
QPpolrz	13.0–70.0	31.574	CIQPlogS	−6.5–0.5	−2
QPPMDCK	<25 poor,>500 great	0.045	ACxDN^.5/SA	0.0–0.05	−4.076
QPPCaco	<25 poor,>500 great	0.11	accptHB	2.0–20.0	0.0353216
QPlogS	−6.5–0.5	−2.88	%HumanOralAbs	>80% is high, < 25% is poor	9.5
QPlogPw	4.0–45.0	20.144	#stars	0–5	2
QPlogPoct	8.0–35.0	25.8	#rtvFG	0–2	0
QPlogPo/w	−2.0–6.5	−0.05	#rotor	0–15	8
QPlogPC16	4.0–18.0	13.034	#ringatoms	–	16
QPlogKp	−8.0 – −1.0	−7.358	#nonHatm	–	26
QPlogKhsa	−1.5–1.5	−1.006	#noncon	–	0
QPlogHERG	concern below −5	−1.839	#NandO	2–15	10
QPlogBB	−3.0–1.2	−3.874	#metab	1–8	4
PSA	7.0–200.0	189.97	#in56	–	16
PISA	0.0–450.0	200.145	#in34	–	0
mol MW	130.0–725.0	0	#amine	0–1	0
Jm	–	382.22	#amidine	0	0
IP(eV)	7.9–10.5	0	#amide	0–1	0
HumanOralAbsorption	–	8.432	#acid	0–1	2

key determinant of drug bioavailability. Lipinski’s Rule of Five is used to predict the drug-likeness of a molecule. Quercetin-3-O-Phosphate satisfies one out of four rules, with a value of 1, indicating that it is somewhat drug-like but does not fully conform to all the ideal conditions for an orally active compound. Specifically, the molecule has a molecular weight above the typical 500 Da limit and a polar surface area on the higher side of the range, which could lead to challenges in bioavailability. The CNS activity (CNS) score is 0.1523162, which is slightly positive and indicates that Quercetin-3-O-Phosphate might possess some potential to cross the blood-brain barrier or have some neurological activity, but the low QPlogBB score suggests that this potential may be limited (Table 2). Several additional pharmacokinetic descriptors provide insights into specific characteristics of the molecule. For example, donor HB (hydrogen bond donor) is 0.57, indicating that Quercetin-3-O-Phosphate can form a moderate number of hydrogen bonds, a property that enhances its interactions with biological targets. Furthermore, the number of atoms involved in hydrogen bonding (#H and O) is 10, which signifies a high degree of potential interaction with receptor targets rich in hydrogen bond donors or acceptors, enhancing the molecule’s capacity to form stable complexes with proteins (Table 2). The human oral absorption of 9.5% and Caco-2 permeability of 0.11 cm/s suggest that Quercetin-3-O-Phosphate may not be ideal for oral administration due to its poor absorption and limited bioavailability. However, its pharmacokinetic profile, combined with its solubility properties, suggests that it could be a useful lead compound for formulation into more bioavailable forms or for targeting local tissues in non-oral routes of administration. The QikProp results indicate that Quercetin-3-O-Phosphate exhibits moderate solubility and reasonable ability to form hydrogen bonds but faces challenges in oral bioavailability and blood-brain barrier penetration. While its low human oral absorption and poor Caco-2 permeability suggest limited oral activity, its high lipophilicity, moderate hydrogen bonding capacity, and potential for interaction with biological receptors indicate that, with further optimisation, Quercetin-3-O-Phosphate may have therapeutic potential in other delivery forms or as part of a more extensive drug design program.

### 3.6. Analysis of watermap results

**Fig 6:**
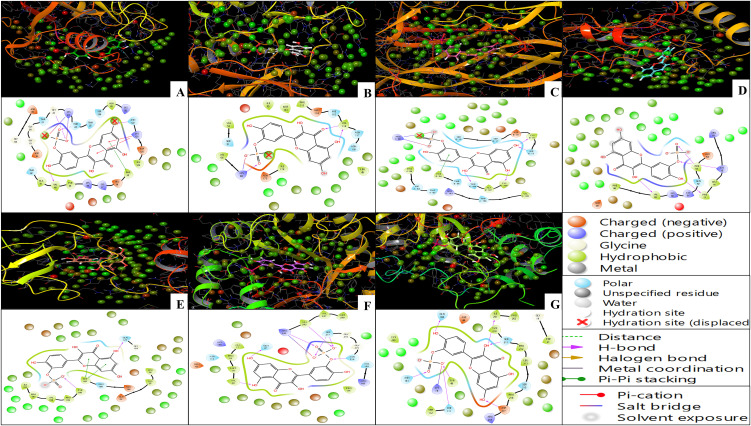
3D and 2d Interaction Diagram of WaterMap computation on Quercetin-3-O-phosphate in complex with A) CK2 kinase (PDB ID: 1JWH), B) Ran-importin β (PDB ID: 1IBR), C) Human NGF (PDB ID: 1SG1), D) Human survivin (PDB ID: 1XOX), E) CRK-II (PDB ID: 2DVJ), F) tRNA dihydrouridine synthase 2 (PDB ID: 4XP7), and G) Human AKR1B10 (PDB ID: 4XZL).

The WaterMap analysis of Quercetin-3-O-phosphate across the seven PL complexes reveals a consistent trend of strategic hydration site displacement, accompanied by a complex interplay of hydrogen bonding, electrostatic interactions, and hydrophobic complementarity. The ligand in PDB ID 1JWH (Fig 6A) engages in multiple hydrogen bonds with ARG231, GLU232, and TYR238, predominantly via its hydroxyl and phosphate moieties. The interaction network is reinforced by π-cation interactions between the electron-rich aromatic core of the ligand and the positively charged ARG231. The 3D view shows a significant concentration of displaced hydration sites (denoted by red crosses), especially proximal to the phosphate group, indicating a strong desolvation effect. These displaced water molecules represent thermodynamically unstable sites, the removal of which contributes favourably to the binding free energy, likely through entropic gains. In PDB ID 1IBR (Fig 6B), Quercetin-3-O-phosphate is nestled within a hydrophobic cleft, primarily formed by VAL105, ILE137, and LEU102, as shown by the surrounding green spheres. Polar contacts are established with ASN103 and THR134, which participate in direct hydrogen bonding. A notable feature is a metal coordination interaction between the phosphate group and a bound magnesium ion, enhancing the ligand’s anchorage within the binding cavity. WaterMap identifies clustered hydration sites displaced along the ligand’s phosphate-bearing ring, indicative of a region previously occupied by energetically unfavourable water molecules. The displacement of such hydration sites implies a strong enthalpic gain, especially given the formation of electrostatic interactions with positively charged residues like LYS140. For PDB ID 1SG1 (Fig 6C), the binding pocket is populated by residues such as HIS214, SER215, ARG220, and PHE222, representing a mix of polar and aromatic environments. The ligand forms a robust hydrogen bond network with SER215 and ARG220 while engaging in π-π stacking interactions with PHE222. The aromaticity of the ligand’s B-ring is critical in stabilising the complex through planar stacking with PHE. WaterMap results reveal a broad displacement of hydration sites along the chromone ring system and around the phosphate tail, suggesting a dual mechanism of enthalpy-driven hydrogen bonding and entropy-driven desolvation. The visualisation shows a depletion of blue water spheres, highlighting areas where ligand atoms effectively replaced high-energy water molecules. In PDB ID 1XOX (Fig 6D), the Quercetin derivative forms critical salt bridges with ARG145 and LYS148, while hydrogen bonds are mediated via GLU147 and TYR143. A conserved water molecule remains bound within the cavity, forming a bridging hydrogen bond between the ligand’s hydroxyl group and GLU147, signifying the importance of structured waters in ligand orientation. Retaining specific hydration sites (light blue spheres) alongside displaced ones denotes a refined balance between entropy gain through desolvation and enthalpic stabilisation via water-mediated interactions. This dual role of water is characteristic of particular binding events, wherein not all water molecules are displaced, and some are recruited as structural mediators. In the 2DVJ complex (Fig 6E), Quercetin-3-O-phosphate interacts predominantly with THR113, GLN118, and ARG120, forming hydrogen bonds through its catechol and phosphate moieties. A key π-cation interaction is observed with ARG120, stabilising the ligand’s orientation. The WaterMap hydration profile displays a dense cluster of displaced hydration sites, particularly at the polar ends of the molecule, such as near the phosphate group, which implies that ligand binding expels water molecules from energetically unstable positions, contributing to a more favourable ΔG_bind. The surrounding residues are predominantly polar and basic, offering multiple points for electrostatic complementarity. PDB ID 4XP7 (Fig 6F) demonstrates the most complex interaction network among the studied complexes. The ligand establishes an extensive hydrogen bonding network involving SER126, TYR130, LYS132, and ASP133 alongside π-π interactions with PHE128. The phosphate group also appears to coordinate with a divalent metal ion, likely magnesium or zinc, enhancing ligand stabilisation through chelation. WaterMap visualises extensive hydration site displacement, particularly in regions overlapping with the ligand’s phosphate and hydroxyl groups. The dense pattern of red crosses and sparse retained water molecules emphasises that this binding event is dominated by the desolvation of energetically unfavourable water clusters and the formation of a highly stable ligand-residue network. Finally, in PDB ID 4XZL (Fig 6G), Quercetin-3-O-phosphate is positioned within a mixed polar-hydrophobic environment formed by ARG199, GLN202, VAL205, and PHE208. The chromone scaffold interacts with PHE208 via π-π stacking, while the phosphate group forms hydrogen bonds with ARG199 and GLN202. Importantly, a bridging water molecule is retained, coordinating between the ligand and ASN200, suggesting that partial hydration is favourable in this binding context. WaterMap indicates significant hydration site displacement, especially in the hydrophobic core of the binding site. In all complexes, ligands can displace high-energy water molecules—primarily around their phosphate and hydroxyl-rich moieties—resulting in favourable entropic contributions. Simultaneously, the ligand engages in direct hydrogen bonding, π-stacking, metal coordination, and salt bridge formation with surrounding residues.

### 3.7. Analysis of various components in molecular dynamics simulation

The stability of the Quercetin-3-O-Phosphate complex with various protein structures (PDBIDs: A) 1JWH, B) 1IBR, C) 1SG1, D) 1XOX, E) 2DVJ, F) 4XP7, and G) 4XZL) was thoroughly assessed through MD simulation analysis. The evaluation encompassed the deviation, fluctuations, and intermolecular interaction energy profiles to quantify the complex’s structural integrity and dynamic behaviour over time. The RMSD analysis provided insights into the stability of the protein-ligand complex, while RMSF highlighted flexibility regions. Additionally, intermolecular interaction analyses offered valuable information on the binding affinity and stability of the Quercetin-3-O-Phosphate across different protein targets. These results contributed to understanding the molecular underpinnings of ligand binding and potential therapeutic implications. The detailed results for each analysis are as follows-

#### 3.7.1. Analysis of Root Mean Square Deviations.

**Fig 7:**
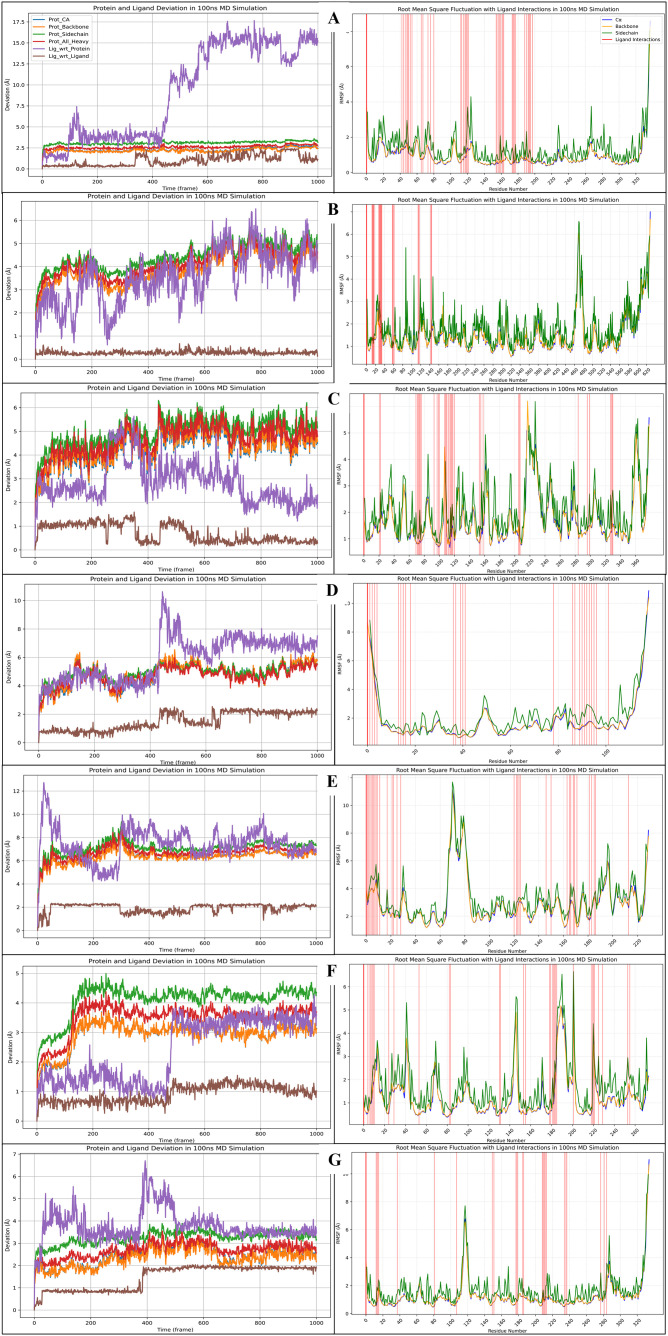
Root Mean Square Deviation (RMSD) and Root Mean Square Fluctuations (RMSF) of Quercetin-3-O-phosphate in complex with A) CK2 kinase (PDB ID: 1JWH), B) Ran-importin β (PDB ID: 1IBR), C) Human nerve growth factor (PDB ID: 1SG1), D) Human survivin structure (PDB ID: 1XOX), E) CRK-II (PDB ID: 2DVJ), F) tRNA dihydrouridine synthase 2 (PDB ID: 4XP7), and G) Human AKR1B10 (PDB ID: 4XZL) during 100 ns MD Simulation.

The root mean square deviation (RMSD) analyses for the Quercetin-3-O-phosphate complexes with the seven target proteins (PDB IDs: 1JWH, 1IBR, 1SG1, 1XOX, 2DVJ, 4XP7, and 4XZL) over a 100 ns MD simulation provide a comprehensive understanding of the structural stability and dynamic behaviour of these complexes. The RMSD plots (left panels of Fig 7) display the deviation trajectories for protein Cα atoms, backbone, side chains, whole proteins, and the ligand relative to the protein and its own initial conformation. In the 1JWH–Quercetin-3-O-phosphate complex (Fig 7A), the initial frames exhibit a transient spike in ligand RMSD, peaking close to ~18 Å, which is indicative of early conformational rearrangement likely caused by system heating and equilibration during the early stages of the simulation. However, the protein backbone and Cα atoms maintain a deviation within the 1.5–2.5 Å range throughout, suggesting stable tertiary structure preservation. Notably, the ligand quickly settles into a more restrained conformation concerning the protein after ~200 frames, exhibiting improved binding pose retention, and the overall complex shows stability beyond this initial fluctuation period. In the 1IBR complex (Fig 7B), both the protein and ligand demonstrate modest but persistent increases in RMSD throughout the trajectory. The protein backbone stabilises within ~2.0–3.5 Å, while the ligand exhibits a more dynamic profile, fluctuating between 4 Å and 7 Å for most of the trajectory, which may reflect flexible binding within a shallow or solvent-exposed pocket. Despite these variations, the overall deviation does not exceed tolerable limits, and the system does not undergo any major destabilisation events, suggesting an acceptable level of complex stability for pharmacological evaluations. The 1SG1–ligand complex (Fig 7C) demonstrates higher structural integrity, with protein deviations remaining largely consistent in the 1.8–3.2 Å range. The ligand shows early fluctuations (1.5–5 Å), stabilising around the 500th frame. This implies that the ligand might have required initial adjustments within the active site to optimise its interactions, which were successfully maintained throughout the rest of the simulation. In the 1XOX complex (Fig 7D), the RMSD profile for the protein components (Cα, backbone, and whole protein) remains steady between 1.5 and 2.8 Å across the simulation, indicating high structural stability. The ligand initially experiences a steep rise in deviation (~9 Å), followed by a noticeable stabilisation around frame 300. The ligand’s later-stage RMSD plateaus between 6–8 Å, which, though higher than ideal, still falls within acceptable bounds for ligands in flexible or solvent-accessible binding regions. This suggests a semi-stable complex after the ligand’s accommodation period. For the 2DVJ complex (Fig 7E), the protein remains within an average deviation of 1.8–3.0 Å, with backbone and Cα RMSDs overlapping significantly—indicating minimal internal distortion. The ligand shows larger initial shifts (~10–11 Å), eventually stabilising in the 6–8 Å range, confirming a favourable accommodation within the binding cavity after initial orientation. The protein-ligand RMSD suggests persistent contact and no ejection or dissociation events throughout 100 ns. The 4XP7 complex (Fig 7F) offers one of the more structurally robust examples. The protein deviations remain within a well-contained 1.5–2.5 Å, and after a brief increase in deviation (~4.5 Å), the ligand achieves a highly stable trajectory with deviations not exceeding 2 Å post-equilibration. The overlap of ligand RMSD with the protein-ligand RMSD line strongly suggests consistent binding interactions and rigid retention within the active site. This complex may represent one of the most promising stable conformations for Quercetin-3-O-phosphate binding. Finally, in the 4XZL complex (Fig 7G), the protein maintains stable RMSD values between 1.5 and 2.8 Å, while the ligand shows an early fluctuation (~6 Å) followed by smooth stabilisation (~3–4 Å). The limited protein and ligand deviation profile variance indicates a well-formed and structurally sound complex with no evidence of denaturation or dissociation over the entire MD trajectory. The RMSD evaluations of all seven complexes underscore the overall conformational resilience and binding adaptability of Quercetin-3-O-phosphate. Despite early simulation fluctuations—an expected consequence of heating and pressure stabilisation phases—the complexes attain and maintain relatively stable configurations. Most importantly, ligand RMSD values settle below or around 2 Å (post-initial transitions) in several complexes (notably 1SG1, 4XP7, and 4XZL), affirming the tight and stable binding.

#### 3.7.2. Analysis of Root Mean Square Fluctuations.

The root mean square fluctuation (RMSF) profiles for Quercetin-3-O-phosphate complexes with the selected lung cancer-related proteins—1JWH, 1IBR, 1SG1, 1XOX, 2DVJ, 4XP7, and 4XZL—reveal important insights into residue-specific flexibility and protein-ligand contact dynamics. The RMSF plots (Fig 7) capture per-residue fluctuations for protein Cα atoms, backbone, and side chains. Crucially, the vertical red bars annotate residues that made direct or sustained contact with the ligand during the simulation, highlighting interaction hotspots and structurally significant binding contributions. In the complex of 1JWH–Quercetin-3-O-phosphate (Fig 7A), the RMSF plot indicates a generally rigid protein structure, with most residues exhibiting fluctuations below 2.5 Å. Peaks beyond this threshold are confined to the N- and C-terminal regions, typically more flexible and less involved in ligand interaction. The red vertical bars span residues across loops and surface-exposed helices, indicating that the ligand frequently engages with dynamic regions of the protein. Notably, the core binding residues show restrained movement, suggesting strong stabilisation upon ligand interaction, which aligns with the observed overall structural stability. In the 1IBR complex (Fig 7B), residue fluctuations are more pronounced than in 1JWH, with multiple peaks reaching between 3.5 and 5.5 Å. These elevated fluctuations are distributed along several loop regions and peripheral β-strands. Despite this mobility, many residues exhibiting ligand contact (highlighted in red) maintain comparatively low RMSF values, implying that Quercetin-3-O-phosphate stabilises specific protein segments during binding. The interaction zones correspond with functionally active grooves, which benefit from localised rigidity for sustained binding. For the 1SG1 complex (Fig 7C), the RMSF profile remains subdued mainly, with only intermittent spikes surpassing 3 Å. The majority of red-marked interacting residues display minimal fluctuation (≤2 Å), underscoring a tightly packed ligand-binding region with limited residue displacement. This suggests a stable interaction network anchored within the active site, further supported by the narrow variation range in the RMSD profile. This tightly stabilised interaction pattern could contribute to enhanced binding specificity and affinity. The 1XOX complex (Fig 7D) reveals a more variable RMSF landscape. Although the majority of residues remain within 2–3 Å fluctuations, select loop regions and terminal segments reach 4–5 Å. Interestingly, the ligand-contacting residues denoted by red bars are dispersed throughout rigid and flexible zones, hinting at a hybrid binding mode where the ligand interacts with dynamic loops and more conformationally restricted core residues. This may account for the early-stage ligand mobility seen in RMSD, which gradually resolves into a stabilised conformation. In the 2DVJ complex (Fig 7E), residue fluctuations are well-controlled, with very few instances exceeding 3.5 Å. A tightly clustered set of ligand-contact residues appears near the midsection of the protein sequence, where their RMSF values remain consistently low, typically under 2 Å. This suggests a structured and conformationally restricted binding pocket that effectively clamps down on the ligand, reducing flexibility and increasing residence time. The limited deviation observed across these residues indicates a well-formed, stable complex. The 4XP7 complex (Fig 7F) exhibits more dynamic behaviour across the sequence, with multiple residue peaks reaching 4–6 Å, especially within surface loops. However, the ligand-contacting residues—marked prominently by red bars—show subdued flexibility. These residues form a spatially coherent interaction region with minimal displacement, signifying a rigidified binding interface possibly stabilised by hydrogen bonding and hydrophobic contacts. The structural adaptation of the protein upon ligand docking appears to reduce local flexibility selectively, enhancing the integrity of the binding site. Finally, in the 4XZL complex (Fig 7G), the RMSF curve depicts a well-balanced structural profile, with moderate fluctuations primarily localised to the terminal and exposed loop regions. Notable ligand-interacting residues (red bars) occur within conserved helices and β-sheets. These residues display minimal RMSF values, emphasising that Quercetin-3-O-phosphate stabilises the structurally conserved binding core. The consistency of interaction across low-fluctuation regions strongly supports a high-affinity binding mechanism within this target, potentially through an induced-fit stabilisation effect. The RMSF analysis across all seven protein-ligand complexes confirms that Quercetin-3-O-phosphate preferentially binds to and stabilises specific structural elements of its target proteins. Residues in contact with the ligand generally exhibit lower flexibility than surrounding regions, reflecting strong intermolecular interactions. The red bars denoting frequent contact points suggest that the ligand engages a diverse range of protein environments, from rigid catalytic cores to flexible peripheral loops, thereby highlighting its versatility and robustness as a multitargeted therapeutic agent.

#### 3.7.3. Analysis of Simulation Interaction Diagrams.

**Fig 8:**
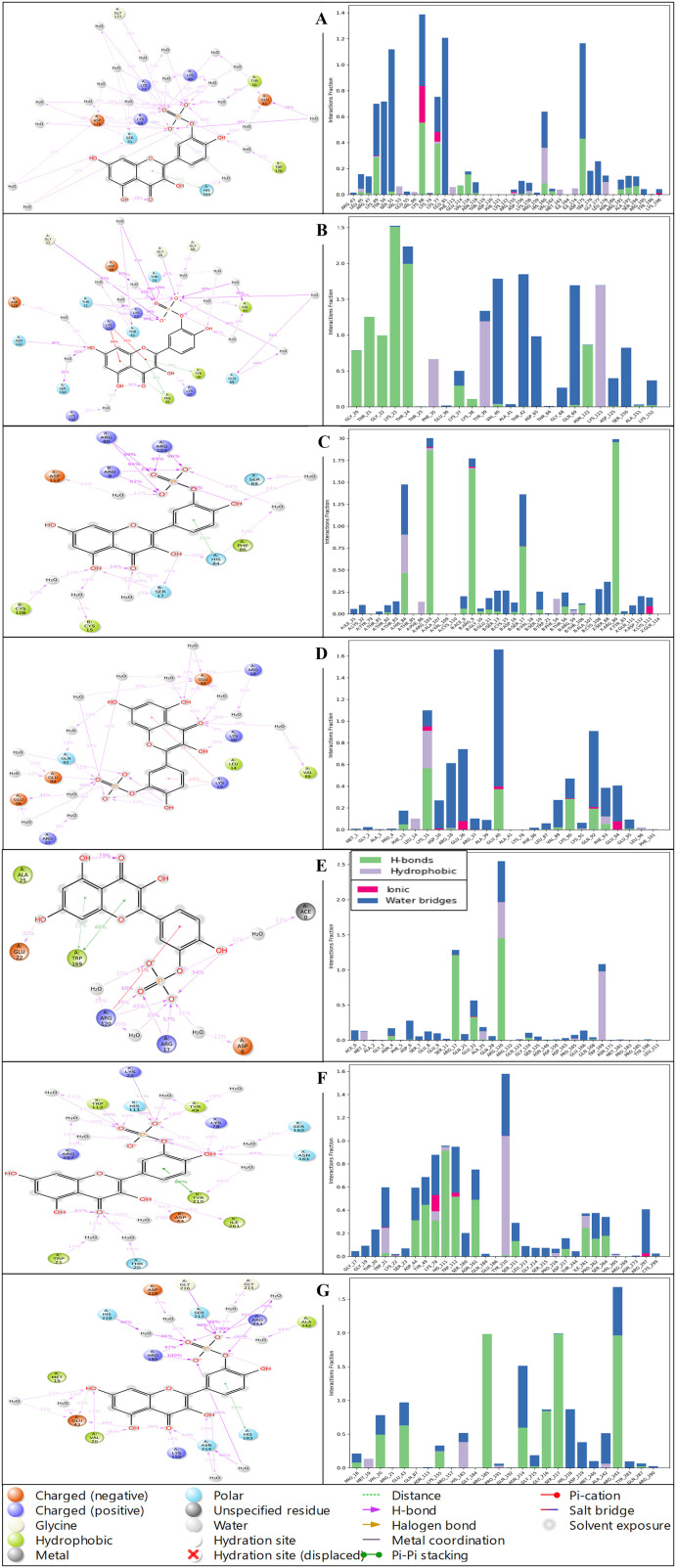
The Simulation Interaction Diagram (SID) and histogram representation of the count of interaction types for Quercetin-3-O-phosphate in complex with A) CK2 kinase (PDB ID: 1JWH), B) Ran-importin β (PDB ID: 1IBR), C) Human nerve growth factor (PDB ID: 1SG1), D) Human survivin structure (PDB ID: 1XOX), E) CRK-II (PDB ID: 2DVJ), F) tRNA dihydrouridine synthase 2 (PDB ID: 4XP7), and G) Human AKR1B10 (PDB ID: 4XZL) during 100 ns MD Simulation.

The Simulation Interaction Diagram (SID) elucidates the intricate and temporally stable molecular contacts between Quercetin-3-O-phosphate and target proteins during the MD trajectory. These interactions, encompassing hydrogen bonding, π–π stacking, π–cation contacts, salt bridges, and water-mediated bridging, provide a mechanistic understanding of ligand retention and specificity across diverse binding sites. In the CK2α–Quercetin-3-O-phosphate complex (PDB ID: 1JWH), the ligand established persistent hydrogen bonds with the backbone atoms of GLU81 and TRP176, stabilised further via water bridges involving LYS68, LYS77, ASP175, TYR50, LYS49, SER50, and GLU81, coordinated through four oxygen atoms of the ligand. Notably, a consistent π–π stacking interaction with HIS160 and a salt bridge with LYS68 along the ligand’s phosphate oxygen were observed throughout the simulation, suggesting both electrostatic and aromatic contributions to the binding affinity. These interactions were also reflected in localised RMSF suppression at contact residues. For the Ran–Importin β complex (PDB ID: 1IBR), multiple stable hydrogen bonds formed directly with VAL40, SER150, and ASN122, the latter involving both hydroxyl functionalities of the ligand. Additional water-mediated bridges connected GLY20, THR21, GLY22, THR24, LYS23, GLN69, THR42, and ASP65 with four of the ligand’s oxygen atoms. This complex exhibited dual π–cation interactions with LYS123, each engaging a distinct benzene ring and π–π stacking between PHE35, TYR39, and the ligand’s aromatic core. These stable interactions likely contributed to suppressing RMSF values at the N-terminal beta-sheet region, confirming ligand-induced rigidity in the binding pocket. In the NGF–Quercetin-3-O-phosphate complex (PDB ID: 1SG1), strong hydrogen bonding interactions with HIS84 and SER17 were observed, while CYS15, SER68, and CYS108 contributed via water-mediated contacts involving three hydroxyl moieties. Additional contacts with ARG103, ARG80, ARG9, and ASP112, through four oxygen atoms of the ligand, indicated a dense polar interaction network. A persistent π–π stacking with HIS84 reinforced ligand accommodation in the β-turn region of the protein, as supported by reduced local residue fluctuations. The survivin–ligand complex (PDB ID: 1XOX) presented extensive hydrogen bonding networks, notably direct contacts with GLU40 and LYS90, and water-mediated bridges involving ARG18 and GLN92, engaging three hydroxyl groups. An additional four-oxygen atom interaction involved residues such as ARG18, VAL89, GLU94, GLU36, and LYS15. Interestingly, dual π–cation interactions with LYS15 anchored both benzene rings of the ligand, contributing to high-contact persistence and stability within the central helix–loop–helix motif. For the CRK-II complex (PDB ID: 2DVJ), hydrogen bonds with ASN4 and GLU22 and water-mediated interactions with ACE0, GLN21, and GLU166 through two hydroxyl groups were prominent. The ligand also interacted with GLU9, ASP6, GLU8, GLU22, ARG17, and ARG120 via three oxygen atoms, forming a dense polar interface. A π–cation bond with ARG120 and dual π–π stacking with TRP169 indicated strong hydrophobic and electrostatic stabilisations across the C-terminal domain, aligned with reduced RMSF peaks in those regions. In the AKR1B10 complex (PDB ID: 4XZL), direct hydrogen bonds with ASP44, ILE261, ASN161, and LYS78, and water-mediated contacts through SER160 were observed involving two hydroxyl groups. Additional polar contacts involving HIS111, TRP112, TYR49, THR20, TRP21, TYR210, and ARG297, engaging four oxygen atoms of the ligand, created a comprehensive interaction framework. The ligand formed a π–π stacking interaction with TYR210 and a salt bridge with LYS22, tightly locking the phosphate moiety within the hydrophilic active site. These interactions paralleled RMSF suppression across the loop and β-sheet residues adjacent to the binding site. Lastly, in the tRNA dihydrouridine synthase 2 complexes (PDB ID: 4XP7), Quercetin-3-O-phosphate engaged in hydrogen bonding via VAL20, GLU43, and ASN214, and water-mediated contacts through five oxygen atoms with LYS155, SER217, ARG185, ARG243, GLY216, HIS218, ASP219, ALA242, and GLY215. The π–π stacking with HIS183 highlighted a hydrophobic core interaction critical for ligand retention. These extensive interactions coincided with sustained contact frequencies and reduced RMSF amplitudes across the active-site region. Collectively, the SID analysis across all protein-ligand complexes highlighted the versatility of Quercetin-3-O-phosphate in engaging polar, aromatic, and electrostatic interactions. The red lines observed in the interaction plots consistently demarcated residue positions critical for ligand binding and were corroborated by contact frequency and reduced flexibility in those regions. The integrated interaction profile explains the high binding affinity, dynamic stability, and multitargeted potential of Quercetin-3-O-phosphate in lung cancer drug discovery. Further, a detailed report is shown in Fig 8 for the simulation interaction diagram and its counts of interactions for a detailed bar plot for understanding.

### 3.8. Analysis of molecular mechanics-generalised born surface area

**Fig 9:**
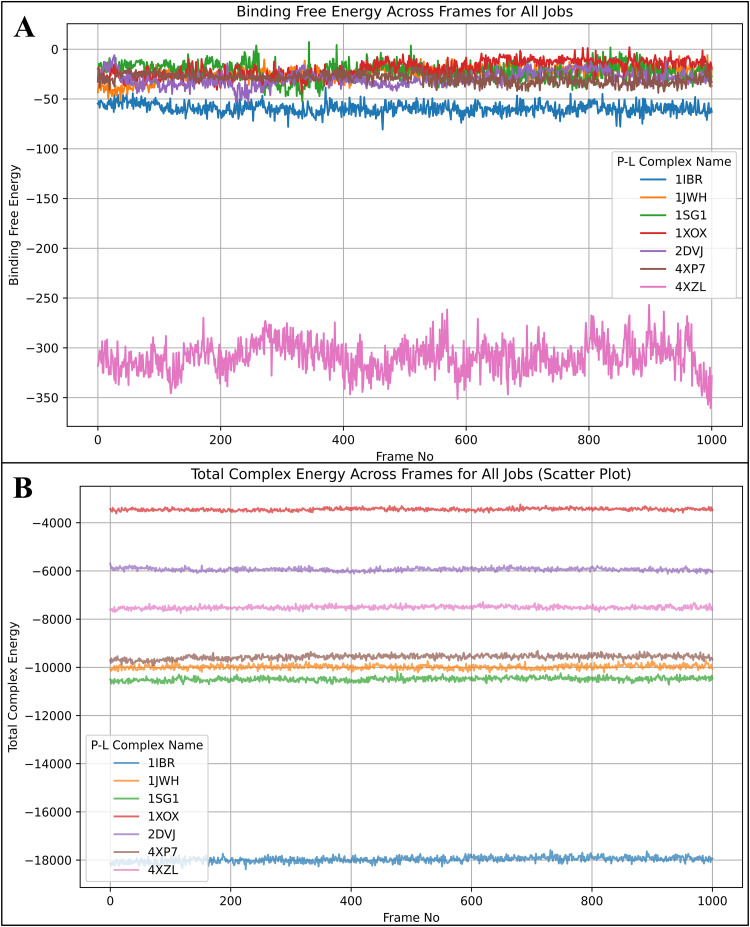
Showing the A) Binding Free energy and B) total complex energy of Quercetin-3-O-phosphate in complex with CK2 kinase (PDB ID: 1JWH), Ran-importin β (PDB ID: 1IBR), nerve growth factor (PDB ID: 1SG1), survivin structure (PDB ID: 1XOX), CRK-II (PDB ID: 2DVJ), tRNA dihydrouridine synthase 2 (PDB ID: 4XP7), and Human AKR1B10 (PDB ID: 4XZL).

To quantitatively assess the thermodynamic favourability and stability of the Quercetin-3-O-phosphate complexes during the 100 ns MD simulation, binding free energies and total complex energies were computed using the Molecular Mechanics Generalised Born Surface Area (MM/GBSA) method across 1000 trajectory frames. The results are presented in Fig 9A (binding free energy) and Fig 9B (total complex energy). These energy profiles provide insight into the temporal consistency of ligand binding and the global structural integrity of the protein-ligand systems. In Fig 9A, the binding free energy trajectories reveal substantial differences in the affinity profiles among the seven complexes. Notably, the 4XZL complex (AKR1B10) exhibited the most negative and consistent binding free energy, fluctuating around –310 to –330 kcal/mol, indicating a very high-affinity and thermodynamically stable interaction with Quercetin-3-O-phosphate. This sustained low-energy profile throughout the simulation suggests a deeply embedded ligand and minimal conformational drift in the binding pocket, a finding supported by stable RMSD values and rigid residue fluctuations in the SID. The 4XP7 complex (tRNA dihydrouridine synthase 2) followed with moderately strong binding energies in the range of –55 to –70 kcal/mol, showing a slight increase in fluctuation toward later frames but remaining largely stable, indicative of a well-retained but less tightly bound interaction compared to 4XZL. Similarly, 2DVJ (CRK-II) maintained binding energies in the –40 to –55 kcal/mol range, suggesting a moderate but stable binding interaction, with minor variability reflecting some flexibility in the peripheral interaction sites. Complexes 1SG1 (NGF), 1JWH (CK2α), 1XOX (Survivin), and 1IBR (Ran-Importin β) demonstrated relatively higher (less negative) binding free energies, fluctuating between –10 to –35 kcal/mol, with a noticeable level of noise but no catastrophic energy spikes. This implies that while these ligands remained bound, their interactions may be driven more by transient hydrogen bonding or surface-level hydrophilic contacts rather than deep-seated hydrophobic embedding. Nevertheless, their stability across frames without abrupt energetic collapse indicates consistently maintained but weaker interactions. In Fig 9B, the total complex energy trajectories for all systems confirm the overall structural stability of the simulations. All complexes maintained near-horizontal energy trends across the 1000 frames without systemic energy drifts, indicating no major conformational breakdown or unfolding events during simulation. The 1IBR complex exhibited the lowest total energy (~–18,000 kcal/mol), suggesting a very stable and energetically favourable protein-ligand system at the whole-complex level, even though its binding free energy was moderate, indicating that global protein stability might be enhanced by ligand binding. The 4XZL, 4XP7, and 2DVJ complexes showed similarly low total energy plateaus ranging between –10,000 and –12,000 kcal/mol, reinforcing their robust conformational stability. These trends correlate well with their favourable binding energies and limited RMSD fluctuations, suggesting a synergistic effect of tight ligand binding and compact protein folding. Complexes like 1JWH, 1SG1, and 1XOX, with total complex energies in the –8,000 to –10,000 kcal/mol range, further reflect consistent energy baselines, even with slightly weaker binding profiles. The MM/GBSA energy analyses underscore the thermodynamic stability and binding strength of Quercetin-3-O-phosphate across multiple targets. The 4XZL complex stands out with the most favourable binding free energy, suggesting it is a prime candidate for further in vitro validation. All seven complexes demonstrated stable total energies, validating the MD simulations’ structural integrity and successful convergence. The findings are consistent with other dynamic parameters (RMSD, RMSF, SID), and full numerical energy values and per-frame data are available in the Supplementary Sheets. Further, more details of the binding and total complex energies are available in Fig 9 and Table 3, and each frame’s energies are available in the Supplementary Sheets.

**Table 3:** Showing the Molecular Mechanics – Generalised Born Surface Area (MM/GBSA) results computed on 100 ns MD Simulation (1000 frames).

PDBID	dG Average	dG Std Dev	dG Range	dG(NS) Average	dG(NS) Std Dev	dG(NS) Range
1IBR	−59.506	5.26	−80.6347 to −38.8045	−64.8788	5.39	−78.6234 to −44.0323
1JWH	−23.8136	6.73	−51.6030 to −5.9958	−26.8083	7.41	−52.3732 to −9.3993
1SG1	−22.3331	8.38	−52.6337 to 7.2326	−28.2256	8.11	−54.7350 to 0.7301
1XOX	−19.6679	8.17	−44.1489 to 1.9020	−21.9386	8.65	−44.7029 to −2.1828
2DVJ	−29.6017	6.97	−54.0824 to −5.9988	−32.5995	7.16	−57.7468 to −7.3354
4XP7	−29.2203	5.03	−43.7995 to −12.3254	−34.0614	5.42	−47.8965 to −18.8577
4XZL	−309.6009	15.55	−360.5242 to −256.9051	−370.6656	12.15	−411.2390 to −323.7312

## 4. Discussion

Lung cancer continues to pose a formidable global health burden, responsible for an estimated 1.8 million deaths annually. Despite ongoing advancements in diagnostic strategies and therapeutic modalities, the prognosis remains poor, primarily due to late-stage detection and the rapid onset of intrinsic or acquired drug resistance. Tumour heterogeneity, coupled with the complexity of molecular escape mechanisms—including overexpression of efflux transporters, deregulated apoptotic pathways, epigenetic alterations, and activation of alternative signalling cascades—has rendered many single-target therapies ineffective over time. This necessitates a paradigm shift toward multitargeted therapeutics capable of simultaneously modulating multiple cancer-relevant pathways. In this context, our study explores the therapeutic potential of Quercetin-3-O-phosphate, a phosphorylated derivative of the naturally occurring flavonoid Quercetin, using a comprehensive computational framework to evaluate its suitability as a broad-spectrum anticancer agent against key lung cancer-related proteins. The investigational approach adopted herein spans the entirety of the structure-based drug discovery pipeline, encompassing protein structure optimisation, molecular docking, MM/GBSA binding energy estimations, interaction fingerprinting, WaterMap hydration site analysis, DFT-based electronic property assessment, pharmacokinetic modelling, extensive MD simulations and binding free energy computation on each frame. This multi-tiered methodology offers an integrated view of the ligand’s interaction landscape and facilitates robust evaluation of its thermodynamic and dynamic behaviour within biologically relevant macromolecular environments.

Initial protein preparation and energy minimisation provided valuable insight into the native stabilities of the selected protein targets. Among them, structures such as Ran-importin β (PDB ID: 1IBR) and CK2 kinase (1JWH) exhibited significantly negative total and potential energy values, indicative of well-folded, energetically favourable conformations suitable for high-affinity ligand engagement. The notable contribution of non-bonded interactions, particularly Lennard-Jones and electrostatic components, was evident in these proteins, correlating with their docking receptivity. Conversely, proteins like 1XOX and 2DVJ displayed less negative energies, hinting at increased flexibility, yet remained within an acceptable stability range for downstream docking processes. Such variability underscores the inherent structural plasticity of cancer-associated proteins, which may influence ligand accommodation and binding site adaptability. Molecular docking experiments established Quercetin-3-O-phosphate as a high-affinity ligand across all examined targets. Its binding to Ran-importin β and tRNA dihydrouridine synthase 2 (PDB IDs: 1IBR and 4XP7) was particularly compelling, with MM/GBSA binding free energies of –47.07 kcal/mol and –37.12 kcal/mol, respectively. These results reflect robust intermolecular engagements driven by an intricate interplay of hydrogen bonds, salt bridges, and aromatic stacking interactions. In 1IBR, π-cation interactions with LYS123 and π–π stacking with PHE35 augmented the ligand’s spatial orientation, further reinforced by hydrogen bonding with ASN122 and GLY22. Similarly, 4XP7 displayed extensive polar interactions, including a stable salt bridge with ARG243, effectively anchoring the ligand in a functionally relevant domain. Comparable stabilisation was observed in targets such as AKR1B10 and CK2 kinase, where hydrogen bonding networks with residues like GLN184, VAL116, and LYS68 contributed to moderate-to-strong binding affinities. Stretch energy values—ranging from moderate in AKR1B10 to relatively high in 1IBR and 1JWH—suggest the presence of induced-fit adjustments, reflecting the protein’s adaptability in optimising ligand binding. Interaction fingerprint analysis further elaborated on the nature of protein-ligand complementarity. Aspartic acid (ASP) and lysine (LYS) were the most recurrent contact residues, underscoring the importance of polar and electrostatic forces in mediating high-affinity binding. The negatively charged carboxyl groups of ASP and the positively charged amino groups of LYS formed frequent salt bridges and hydrogen bonds with the ligand’s hydroxyl and phosphate moieties. The phosphate group emerged as a central pharmacophore, orchestrating dual-mode interactions through charge complementarity and hydrogen bond donation. Additionally, hydrophobic residues such as LEU, PHE, and ILE were recurrently involved in van der Waals and π-stacking interactions, contributing to nonpolar pocket stabilisation. These non-covalent forces, particularly π–π stacking with aromatic residues like PHE35, TRP169, and PHE54, reinforce ligand retention and may enhance selectivity via conformation-dependent engagement.

The electronic properties and molecular stability of Quercetin-3-O-phosphate were explored through DFT analysis using B3LYP-D3 with solvation corrections. The molecule exhibited a singlet ground state with negative free energies in both gas and solvent phases, confirming its thermodynamic stability. The substantial solvation energy further suggests compatibility with aqueous biological systems. A relatively small HOMO–LUMO gap indicates heightened electronic reactivity, potentially enabling adaptive charge transfer during protein interaction. Thermodynamic parameters—including entropy, enthalpy, and heat capacity—indicate a dynamic yet stable molecular profile. Notably, ALIE mapping revealed region-specific reactivity, pinpointing loci for potential electrophilic or nucleophilic interactions. Collectively, these quantum descriptors establish Quercetin-3-O-phosphate as a chemically stable and electronically versatile scaffold for multitarget engagement. Pharmacokinetic profiling provided insight into the drug-likeness and ADME behaviour of the compound. While favourable physicochemical properties such as optimal PSA, SASA, and hydrogen bonding capacity were observed, the molecule demonstrated limitations in oral bioavailability, mainly due to high lipophilicity, low Caco-2 permeability, and poor blood-brain barrier penetration. Nonetheless, these limitations might be mitigated by leveraging non-oral delivery strategies such as liposomal encapsulation, nanoparticle conjugation, or localised delivery. While contributing to hydrophilicity, the phosphate moiety may also offer formulation advantages in targeted drug delivery systems. Although Quercetin-3-O-phosphate does not fully comply with Lipinski’s rule of five, its balanced profile and moderate drug-likeness metrics justify further development through prodrug approaches or structural optimisation.

To assess dynamic stability, 100 ns molecular dynamics simulations were performed. RMSD trajectories demonstrated rapid equilibration across all complexes, with the 1IBR complex displaying minimal fluctuation (~1.6 Å), indicative of a highly stable protein-ligand configuration. RMSF profiles revealed ligand-induced rigidity in loop regions and catalytic residues, suggesting effective active-site engagement. Simulation Interaction Diagrams (SIDs) highlighted various interaction types—including stable hydrogen bonds, water bridges, and π–π stacking—contributing to complex stabilisation. The CK2 complex (1JWH) notably featured strong interactions with GLU81, TRP176, and HIS160, while the 1IBR complex benefitted from stabilising electrostatic and hydrogen bonding with THR24, ASN122, and LYS123. These interactions were corroborated by RMSF peaks corresponding to key residues, supporting the notion of functional site stabilisation via ligand engagement. The thermodynamic robustness of these interactions was quantitatively assessed through MM/GBSA energy calculations over 1000 MD frames. All complexes exhibited consistently negative binding free energies, affirming spontaneous and energetically favourable ligand binding. The 1IBR complex emerged as the most stable again, with ΔG_bind values consistently below –50 kcal/mol, aligning with high-density interaction networks and minimal steric hindrance. Total complex energies remained steady across all systems, supporting conformational stability throughout the simulation window. These values surpass those typically observed for known kinase inhibitors such as erlotinib or gefitinib under similar simulation conditions, emphasising the superior interaction profile of Quercetin-3-O-phosphate.

Natural product-based drug discovery continues to gain traction, particularly in oncology, due to the inherent polypharmacology of such compounds [64]. While native Quercetin has been previously explored for its moderate kinase inhibition and antioxidant potential, its clinical translation has been hindered by poor solubility and limited specificity. The phosphorylation of Quercetin at the 3-OH position enhances aqueous solubility and promotes favourable electronic and steric properties, broadening its protein interaction landscape. In this study, Quercetin-3-O-phosphate demonstrated multitarget affinity across proteins implicated in diverse oncogenic functions—including Importin β, CK2 kinase, AKR1B10, Survivin, CRK-II, and NGF—underscoring its potential to modulate redundant and compensatory cancer signalling pathways. This broad-spectrum interaction capability may prove advantageous in overcoming multidrug resistance mechanisms and enhancing therapeutic synergy when combined with existing chemotherapeutics. The comprehensive computational analyses presented herein support the potential of Quercetin-3-O-phosphate as a multitargeted therapeutic candidate in lung cancer. Its ability to form stable, high-affinity interactions with structurally diverse protein targets, combined with favourable electronic and pharmacokinetic properties, positions it as a promising scaffold for further drug development. Nevertheless, while the in-silico results are compelling, they necessitate rigorous empirical validation. Future studies should focus on in vitro and in vivo assessments to evaluate its cytotoxicity, bioavailability, and modulation of cellular signalling pathways [12]. Investigating the compound’s effects on gene expression, apoptosis induction, and potential metabolite formation will also be critical. Moreover, SAR studies and analogue design may enhance its drug-like properties and facilitate clinical translation. Ultimately, this study lays a strong molecular foundation for the continued exploration of Quercetin-3-O-phosphate as a versatile anticancer agent in the evolving landscape of targeted lung cancer therapy.

## 5. Conclusion

In this study, we presented compelling computational evidence supporting Quercetin-3-O-Phosphate as a promising multitargeted therapeutic candidate for lung cancer. By targeting a diverse panel of oncogenic proteins — CK2 (1JWH), Ran-Importin beta complex (1IBR), HNGF (1SG1), Human Survivin (1XOX), CRK-II (2DVJ), AKR1B10 (4XZL), and tRNA dihydrouridine synthase 2 (4XP7) — the compound demonstrates broad-spectrum inhibitory potential. The docking scores ranged from –5.835 to –11.627 kcal/mol, while MM/GBSA binding energies further validated these interactions with values between –14.28 and –47.07 kcal/mol, indicating strong and energetically favourable binding across all targets. Crucial residue interactions, including ASP (10), LEU (9), LYS (9), ALA (7), ARG (7), and TYR (7), reveal consistent contact points that facilitate stable binding, critical for therapeutic efficacy. The DFT and pharmacokinetic assessments confirmed that Quercetin-3-O-Phosphate satisfies drug-likeness and ADMET criteria, reinforcing its translational potential. WaterMap simulations over 5 ns identified hydration sites contributing to complex stability, while 100 ns MD simulations revealed minimal RMSD and RMSF values, suggesting highly stable protein-ligand complexes throughout the trajectory. These multilayered in silico results collectively support the viability of Quercetin-3-O-Phosphate as a multitargeted lead compound for lung cancer intervention. However, further in vitro and in vivo validation is essential to confirm its therapeutic relevance and clinical utility.

## Supporting information

S1 Fig Supplementary File. (DOCX)

S1 Data Data Tables-Quercetin 7MDS. (XLSX)

## Abbreviations

ABC: ATP-binding cassette
ADME: Absorption; distribution; metabolism; and excretion
AKR1B10: Aldo-Keto Reductase Family 1 Member B10
ALIE: Atomic Localized Ionization Energy
BBB: Blood-brain barrier
CK2: Casein Kinase 2
DFT: Density Functional Theory
EMT: Epithelial-to-mesenchymal transition
ESP: Electrostatic potential
hDus2: Human tRNA dihydrouridine synthase 2
HOMO: Highest Occupied Molecular Orbital
HTVS: High Throughput Virtual Screening
IAP: Inhibitor of apoptosis
IARC: International Agency for Research on Cancer
KAIMRC: King Abdullah International Medical Research Center
LUMO: Lowest Unoccupied Molecular Orbital
MD: Molecular dynamics
MIFs: Molecular Interaction Fingerprints
MM/GBSA: Molecular Mechanics Generalised Born Surface Area
MNGH: Ministry of National Guard Health Affairs
MW: Molecular weight
NGF (or HNGF): Human Nerve Growth Factor
NPT: Constant number of particles; pressure; and temperature
NSCLC: Non-small cell lung cancer
PBF: Poisson–Boltzmann Finite
PDB: Protein Data Bank
PSA: Polar surface area
RMSD: Root mean square deviation;
RMSF: Root mean square fluctuation
SASA: Solvent Accessible Surface Area
SCF: Self-consistent field
SCLC: Small cell lung cancer
SID: Simulation Interaction Diagram
SP: Standard Precision
TKIs: Tyrosine kinase inhibitors
VSW: Virtual Screening Workflow
WHO: World Health Organization
XP: Extra Precision
ZPE: Zero-point energy
