# Supplementary material for: Heptapharmacological activity of Quercetin-3-O-phosphate against lung cancer pathways
Source: PLoS One. 2026 Jul 10;21(7):e0349042. doi: 10.1371/journal.pone.0349042 (PMC13353998; doi:10.1371/journal.pone.0349042)
Supplement: S1 Fig — (DOCX) [file pone.0349042.s001.docx]

**Protein–ligand interaction analysis of Control Drug Alectinib (DB11363):**

The molecular interaction profiles of Alectinib (DrugBank ID: DB11363) with seven protein targets were analysed using the two-dimensional interaction diagrams shown in the figure. The diagrams illustrate the residues surrounding the ligand and the types of intermolecular contacts, including hydrogen bonds, π–π stacking, π–cation interactions, salt bridges, hydrophobic contacts and solvent exposure. These interactions collectively contribute to the stability and orientation of the ligand within the binding pockets of the respective proteins. Alectinib demonstrates stable binding within the catalytic pocket of **CK2 kinase (PDB ID: 1JWH)** through a combination of hydrogen bonding, electrostatic interactions and hydrophobic contacts. A hydrogen bond interaction is observed with Lys134, which contributes significantly to the anchoring of the ligand within the ATP-binding pocket. Additional polar stabilisation is provided by Thr257. Negatively charged residues such as Asp426 contribute to electrostatic interactions in the vicinity of the ligand, while Lys346 participates in a π–cation interaction with the aromatic system of Alectinib. The binding pocket is further stabilised by hydrophobic contacts with residues including Val350, Met353, Leu354, Leu85 and Ile95, which form a non-polar environment around the ligand scaffold. Aromatic interactions with Trp176 further strengthen the binding through π–π stacking. The presence of structured water molecules and hydration sites near residues such as Asp339 and Asp340 indicates potential water-mediated stabilisation, while a portion of the ligand remains partially solvent exposed near the entrance of the pocket.

Within the **Ran–importin β complex (PDB ID: 1IBR)**, Alectinib occupies a groove formed by both polar and hydrophobic residues. Hydrogen bond interactions are observed with Asp120 and Asn117, which help stabilise the orientation of the ligand in the binding region. The positively charged Lys122 contributes to electrostatic stabilisation and forms a salt bridge with polar moieties of the ligand. Hydrophobic residues including Val16, Met163, Phe121, Ile95 and Val66 surround the ligand and create a hydrophobic environment that favours the accommodation of the aromatic rings of Alectinib. Additional polar interactions involving His115 and His160 further contribute to ligand stabilisation. Several nearby water molecules suggest the presence of hydration sites that may facilitate indirect or water-mediated contacts between the ligand and protein residues. The interaction of Alectinib with **human nerve growth factor (PDB ID: 1SG1)** occurs within a shallow surface pocket characterised by both charged and polar residues. A hydrogen bond interaction with Glu11 provides a key stabilising contact that anchors the ligand. Arg9 forms a π–cation interaction with the aromatic ring system of Alectinib, thereby strengthening ligand binding. Hydrophobic interactions involving residues such as Pro61, Tyr79 and Cys80 contribute to van der Waals stabilisation within the pocket. Additional polar contacts with Thr81, Thr82 and Ser113 further enhance the stability of the complex through weak hydrogen bonding and dipole interactions. A portion of the ligand remains exposed to the solvent, indicating that the binding region is relatively open and accessible.

In the case of **human survivin (PDB ID: 1XOX)**, Alectinib binds within a pocket composed of both charged and hydrophobic residues. A hydrogen bond interaction is formed with Asp16, which plays a crucial role in stabilising the ligand orientation within the binding site. Lys15 contributes to electrostatic stabilisation through the formation of a salt bridge with polar groups on the ligand. Hydrophobic residues including Leu14, Phe93, Val53 and Leu45 provide a non-polar environment that stabilises the aromatic scaffold of Alectinib through van der Waals interactions. Additional contacts involving Gly46 and Pro4 contribute to ligand stabilisation through backbone interactions. Similar to other complexes, the ligand exhibits partial solvent exposure near the entrance of the binding pocket. The interaction of Alectinib with **CRK-II (PDB ID: 2DVJ)** involves several charged and polar residues within the SH2 domain region. Hydrogen bonding interactions are observed with Glu166 and Gln168, which help maintain the ligand within the binding cavity. Electrostatic interactions involving Arg120 and Arg122 contribute additional stabilisation, with the aromatic rings of Alectinib participating in π–cation interactions with these positively charged residues. Hydrophobic contacts with residues such as Leu18, Leu26, Ala23, Ala25 and Trp15 form a stabilising hydrophobic environment around the ligand. Additional polar residues including Ser125 and Gln123 further support ligand binding through weak hydrogen bonding interactions.

In **tRNA dihydrouridine synthase 2 (PDB ID: 4XP7)**, Alectinib binds close to the catalytic region of the enzyme. A hydrogen bond interaction involving Gly126 contributes to ligand stabilisation within the binding pocket. Electrostatic interactions with Glu166 further enhance the binding affinity of the ligand. The surrounding hydrophobic environment is formed by residues such as Leu26, Ala23, Val48, Phe123 and Trp21, which provide favourable van der Waals interactions with the aromatic structure of Alectinib. Polar residues including Ser125 and Gln123 also participate in stabilising the ligand through additional polar contacts. The presence of several hydration sites suggests that water-mediated interactions may also contribute to the overall stability of the protein–ligand complex. Finally, Alectinib exhibits stable binding within the catalytic cavity of **human AKR1B10 (PDB ID: 4XZL)**. A hydrogen bond interaction with Arg28 plays a significant role in anchoring the ligand within the active site. Asp70 contributes to electrostatic stabilisation through salt bridge interactions with polar groups of the ligand. Hydrophobic residues including Val72, Val73, Leu25 and Phe74 surround the ligand and provide extensive van der Waals contacts. Aromatic stacking interactions with Phe74 further stabilise the complex through π–π interactions. Additional polar interactions involving residues such as Asn276, Thr24 and Thr279 contribute to the overall stability and orientation of the ligand within the binding pocket.

Overall, the interaction analysis demonstrates that Alectinib forms stable complexes with all investigated proteins through a combination of hydrogen bonding, electrostatic interactions, hydrophobic contacts and aromatic stacking. However, the docking scores (supplementary sheets) and the ligand direct interactions are way less than the identified molecule Quercetin-3-O-phosphate in complex*.*


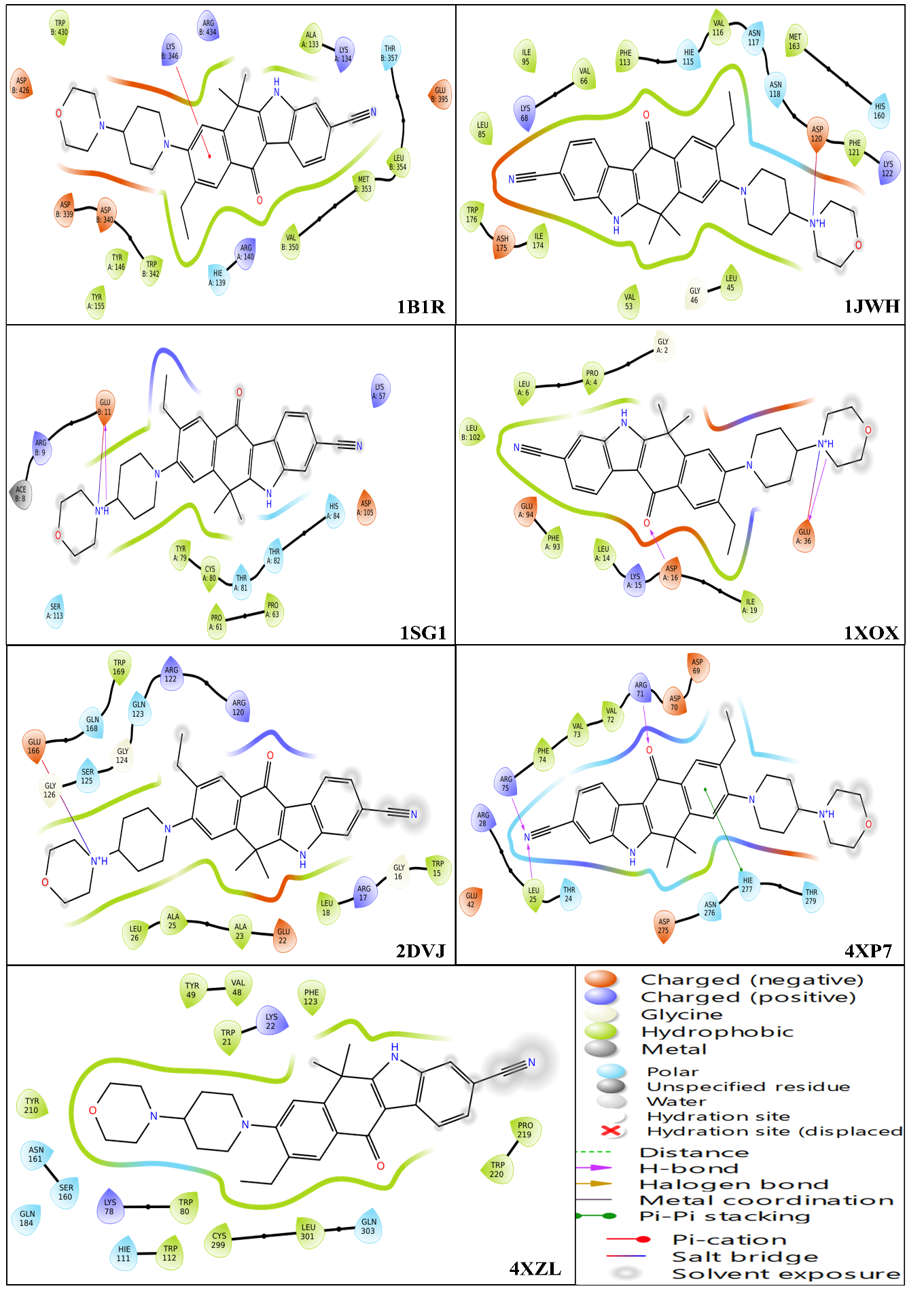


***Supplementary Figure 1.*** *2D ligand interaction diagrams of Alectinib (DB11363) in complex with CK2 kinase (PDB ID: 1JWH), Ran–importin β (PDB ID: 1IBR), human nerve growth factor (PDB ID: 1SG1), human survivin structure (PDB ID: 1XOX), CRK-II (PDB ID: 2DVJ), tRNA dihydrouridine synthase 2 (PDB ID: 4XP7), and human AKR1B10 (PDB ID: 4XZL). The diagrams illustrate the key amino acid residues and intermolecular interactions involved in stabilising Alectinib within the binding pockets of the respective proteins.*
